# Aptamer-Hytac Chimeras for Targeted Degradation of SARS-CoV-2 Spike-1

**DOI:** 10.3390/cells13211767

**Published:** 2024-10-25

**Authors:** Carme Fàbrega, Núria Gallisà-Suñé, Alice Zuin, Juan Sebastián Ruíz, Bernat Coll-Martínez, Gemma Fabriàs, Ramon Eritja, Bernat Crosas

**Affiliations:** 1 Department of Surfactants and Nanobiotechnology, Institute for Advanced Chemistry of Catalonia (IQAC-CSIC), Jordi Girona 18, 08034 Barcelona, Spain; carme.fabrega@iqac.csic.es; 2 Centro de Investigación Biomédica en Red de Bioingeniería, Biomateriales y Nanomedicina (CIBER-BBN), 28029 Madrid, Spain; 3 Proteasome Regulation Lab, Department of Cells and Tissues, Molecular Biology Institute of Barcelona (IBMB-CSIC), Baldiri i Reixac 4, 08028 Barcelona, Spainazubmc@ibmb.csic.es (A.Z.);; 4 Lincbiotech SL, Avenida do Mestre Mateo, 2, 15706 Santiago de Compostela, Spain; jruiz@lincbiotech.com; 5 Department of Biological Chemistry, Institute for Advanced Chemistry of Catalonia (IQAC-CSIC), Jordi Girona 18, 08034 Barcelona, Spain; gemma.fabrias@iqac.csic.es

**Keywords:** coronavirus, SARS-CoV-2, spike protein, aptamer, oligonucleotide, adamantyl, tert-butyl carbamate (Boc), targeted protein degradation

## Abstract

The development of novel tools to tackle viral processes has become a central focus in global health, during the COVID-19 pandemic. The spike protein is currently one of the main SARS-CoV-2 targets, owing to its key roles in infectivity and virion formation. In this context, exploring innovative strategies to block the activity of essential factors of SARS-CoV-2, such as spike proteins, will strengthen the capacity to respond to current and future threats. In the present work, we developed and tested novel bispecific molecules that encompass: (i) oligonucleotide aptamers S901 and S702, which bind to the spike protein through its S1 domain, and (ii) hydrophobic tags, such as adamantane and tert-butyl-carbamate-based ligands. Hydrophobic tags have the capacity to trigger the degradation of targets recruited in the context of a proteolytic chimera by activating quality control pathways. We observed that S901-adamantyl conjugates promote the degradation of the S1 spike domain, stably expressed in human cells by genomic insertion. These results highlight the suitability of aptamers as target-recognition molecules and the robustness of protein quality control pathways triggered by hydrophobic signals, and place aptamer-Hytacs as promising tools for counteracting coronavirus progression in human cells.

## 1. Introduction

Severe acute respiratory syndrome coronavirus 2, also known as SARS-CoV-2, is the virus causing the respiratory syndrome known as COVID-19 and the consequent worldwide pandemic [1,2]. SARS-CoV-2 is an enveloped coronavirus belonging to the betacoronavirus genus, together with Middle East respiratory syndrome coronavirus (MERS-CoV) and SARS-CoV [1]. Viral particles consist of a lipidic envelope integrating membrane protein (M), nucleocapsid protein (N), envelope protein (E), and spike protein (Spike or S). M protein is the main protein constituent in coronavirus, and it is thought to give virions their shape. E protein, which has ion channel activity, is present in smaller quantities and takes part in viral assembly and release. N protein is responsible for packing viral genomic gRNA.

Spike plays a key role in virus–human cell interaction, by means of the recognition of human ACE2 cellular receptors [3]. Spike is divided into two regions, S1 and S2. S1, which covers amino acid positions 1-685, contains the N-terminal domain (NTD), the receptor binding domain (RBD), and the subdomains 1 and 2 (SD1 and SD2). S2, which covers amino acid positions 686-1273, contains the following regions: peptide (FP), heptad repeat 1 (HR1), central helix (CH), connector domain (CD), heptad repeat 2 (HR2), transmembrane domain (TM), and cytoplasmic tail (CT). The spike protein is processed by cellular transmembrane serine protease 2 (TMPRSS2) and the resulting cleaved form inserts itself into the host’s membrane, anchoring the virus and helping in the membrane fusion. This process permits the early entrance of N-protein-packed viral RNA into the cell, where the 29.9 kb positive single strand +RNA genome starts to translate, initiating a virion production cycle. Thus, Spike is an essential constituent of the virion in order to be infective. Therefore, it has become a key pharmacological target, with multiple vaccine and antiviral strategies focused on Spike [4].

Recent years have consolidated proximity-inducing pharmacology by means of bispecific agents as a powerful strategy [5]. This vast approach is mainly based on chimeric compounds that put together two interactive moieties that concurrently bind to a target and an effector [6]. Within this field, proteolysis-targeting chimeras (Protacs) occupy an advanced position, due to their quick development and therapeutic potential, initially focused on cancer, but more recently expanding to other pathologies [6,7]. Protacs are typically based on the structural frame that includes: (i) ligands that engage distinct E3 ligases (mainly, cereblon (CRBN) and von Hippel–Lindau (VHL)), (ii) target-recruiting ligands, (iii) and different lengths and types of linkers [6,8]. Beyond Protacs, different types of proteolytic chimeras have emerged in the last years, in an attempt to complement mechanisms of action and provide alternative tools, demonstrating that different types of ligands, distinct proteolysis signals, and pathways can be exploited for this purpose. Thus, for instance, chimeras promoting autophagy or targeting protein cargos to the lysosome by means of endocytic events have been reported [8,9,10,11].

Proteolytic chimeras based on hydrophobic tags (Hytacs) have been developed. The presence of a hydrophobic tag within a chimera triggers the unfolded protein response (UPR) upon the formation of the protein–ligand complex. Thus, the exposure of hydrophobic residues to the solvent may be recognized by molecular chaperones as a signal of a misfolded protein. These chaperones ultimately promote target degradation by the proteasome when hydrophobic motifs persist. Different hydrophobic tags have been described to produce efficient target degradation when used as ligands linked to a target-binding compound: adamantane [12], Boc_3_-arginine (Boc_3_-Arg) [13], pyrene [14], fluorene [15], carborane [16], menthoxyacetyl [17], and norbornene [18].

In the case of adamantyl, the rigid and hydrophobic group has been linked to several protein ligands and applied to degrade different targets. One of the most successful applications of adamantyl-based chimeras is the development of androgen receptor degraders, compounds applied to androgen-dependent cancer cells [19]. Moreover, pseudokinase Her3 has been efficiently degraded with the adamantane ligand TX2-121-1 [20]. Application of the Boc_3_-Arg hydrophobic tag has been reported in trimethoprim to target dihydrofolate reductase [21], as well as in modified diuretic drugs, such as the ethacrynic acid derivative EA-B_3_A, and other ligands designed to target glutathione-S-transferases [13,22]. An additional application of the Boc_3_-Arg-ligand is shown in chimeras targeting proprotein convertase subtilisin-like/kexin type 9 (PCSK9), a serine protease involved in a protein–protein interaction with a low-density lipoprotein (LDL) receptor. Inhibiting PCSK9 decreases LDL cholesterol levels, which has a high potential for anti-atherosclerosis therapy. The Merck group developed a set of PCSK9 small-molecule binders in order to create specific Protacs. In this work, Protacs based on E3-ligases ligands did not induce degradation; instead, the Boc_3_Arg-ligand could induce a significant decrease in PCSK9 endogenous levels [23]. Overall, degradation chimeras exhibit high design flexibility, not only among the ligands that recruit effectors, but also in target-binding ligands, which may be based on different types of compounds, including small molecules, peptides, antibodies, and aptamers [6].

Aptamers are single-stranded oligonucleotides, based on DNA or RNA that adopt complex three-dimensional structures, including internal conformations of rings, hairpins, stems, and others [24,25,26]. Aptamers exhibit features that make them highly valuable tools in targeted pharmacology and, more specifically, in targeted protein degradation (TPD) strategies. Aptamers may bind to the target protein with high specificity and affinity [27], offering high solubility, low hydrophobicity, good tissue penetration, and cell internalization. Synthesis of aptamers via DNA solid phase methods facilitates large-scale production. In addition, they can be chemically modified and exhibit low immunogenicity. Therefore, aptamers are a type of ligands that offer advantages with respect to other surface-recognizing ligands. Moreover, aptamers are widely used, not only in therapeutics, but also as tools in nanotechnology (diagnostic nano-detectors, drug-deliverers biosensors, nanocarriers) [28,29,30].

The capacity of aptamers to bind to surfaces and motifs of proteins is defined as a mechanism of action with a high applicability in medicine and nanotechnology [25]. Several aptamers targeting disease-related proteins for degradation have been developed in the last years, illustrating their high adaptability and potential [28,31]. The oligonucleotide AS1411 (formerly AGRO100), which was earlier defined as a nucleolin binder with antiproliferative activity, is a 26-mer oligodeoxynucleotide that contains only guanines and thymines and in solution forms a guanine-quartet-mediated dimer [32]. AS1411, which triggers the internalization of cell-surface nucleolin and also binds to cytoplasmic nucleolin, inhibits cell proliferation in a wide range of cancer cell lines [33]. Furthermore, the destabilization of B-cell lymphoma protein 2 (BCL-2) mRNA [34], and the inhibition of nuclear factor-κB [33], promoted by AS1411, have been also described. Notably, chimeras based on the aptamer AS1411 and inducing the degradation of nucleolin have been developed. Nucleolin, which is highly expressed in tumor cells and used as a biomarker for anti-tumor therapy, appears as a suitable target for a TPD approach. Zhang and collaborators designed an aptamer-based Protac using AS1411 linked to the VHL ligand AHPC. With this approach, nucleolin was efficiently degraded in breast cancer cells in a highly selective manner [35].

Aptamers interacting with the estrogen receptor-alpha (ER*α*) conjugated with well-defined ligands for CRBN, VHL, and cIAP E3-ligases have been also developed. These aptamers were tested for ER*α* degradation capacity through assays in which conjugated aptamers were internalized with transfection reagents. They observed ER degradation in the presence of aptamer conjugates, with the highest activity exhibited by the aptamers signalized to CRBN [36].

In an additional recent report, Tian and collaborators developed a novel type of aptamer-based chimeras, the insulin-like growth factor 2 (IGF2)-tagged aptamer chimeras (ITACs). This novel type of degrader enables targeted degradation of cell-surface proteins leveraging IGF2 internalization. Thus, ITACs are composed of two modules: IGF2 for binding to cell-surface lysosome-shuttling receptor insulin-like growth factor 2 receptor (IGF2R), and aptamers for recognizing protein targets to degrade. As a proof-of-concept, the authors selected the tyrosine-protein kinase cellular mesenchymal-epithelial transition factor (*c*-MET), due to its role in cancer proliferation, invasion, and metastasis, and its high abundance. To assess the versatility of this approach, dual chimeras linking the protein tyrosine kinase 7 (PTK7) with IGF2R were also tested and validated for PTK7 degradation. Furthermore, the authors developed a trivalent version of ITACs, which concurrently interacted with two plasma membrane POIs (in this instance, *c*-MET and PTK7) and with IGF2R. Degradation of both POIs with equal efficiency, was observed [37].

Moreover, aptamer-Lytacs designed for the degradation of extracellular and plasma membrane proteins have been developed [38,39]. This type of chimeras is based on trivalent N-acetyl-galactosamine (tri-GalNAc), which avidly binds to the asialoglycoprotein receptor (ASGPR), a ligand used for the degradation of membrane proteins and extracellular proteins in liver, when conjugated to antibodies or other ligands [38,39].

In the present work, we explored aptamer-Hytac chimeras for targeted degradation of SARS-CoV-2 Spike. For this purpose, we conjugated S1-binding aptamers to adamantyl and Boc_3_-Arg hydrophobic ligands and tested the resulting conjugates for degradation of S1 protein expressed in human cells. Moreover, we generated human cell lines stably expressing S1-domain, and used them for testing S1 degradation by setting up a benchmark protocol for aptamer-Hytac characterization. We observed that, at 100–200 nM concentrations, aptamer–adamantyl chimeras could differentially decrease S1 protein levels in human cells. These observations make conceivable the use of chimeras based on aptamers targeting viral proteins as proteolytic agents. Furthermore, the use of hydrophobic ligands, such as the adamantyl group, opens the possibility of exploiting a mechanism of action that relies on robust quality control pathways, such as unfolded protein response. By putting together the high affinity of aptamers and the robustness of degradation induced by Hytacs, we generated a proof-of-principle of chimeras that may act in the degradation of intracellularly expressed viral proteins.

## 2. Materials and Methods

### 2.1. Oligonucleotides and Aptamers Preparation

The aptamers S901 and S702 carrying biotin at the 3′end used in ELONA experiments were purchased from Biomers and used directly as received. The synthesis of Hytacs conjugates was performed with oligonucleotides carrying an alkylamino group at the 5′-position. A short oligonucleotide sequence (5′-aminohexyl-T_8_) was prepared in our laboratory using monomethoxytrityl (MMT)-6-aminohexyl phosphoramidite for the incorporation of an alkylamino group at the 5′end. This oligonucleotide was used to set up the optimal conditions for the preparation of Hytacs conjugates. Then, 5′-aminohexyl-aptamers S901 and S702 with or without biotin at the 3′-end were purchased to Biomers on a 1 µmol scale. 5′-aminohexyl-S901-3′-Cy5 oligonucleotide was synthesized in our laboratory on a 1 µmol scale using the commercially available solid support functionalized with cyanine 5 and the MMT-6-aminohexyl phosphoramidite. To avoid Cy5 degradation, G was protected with the dimethylformamidino (dmf) group that can be removed at room temperature. All the oligonucleotides carrying 5′amino groups were stored attached to the controlled pore glass (CPG) support and with the MMT group on. A small amount of the solid supports containing each one of the aptamers and the NH_2_-T_8_ sequence was treated with 3% trichloroacetic acid (TCA) in dichloromethane (DCM) solution to remove the MMT group and deprotected with 32% aqueous ammonia overnight at 65 °C (5′-amino-aptamers) or 2 h at room temperature (amino-T_8_), respectively, to obtain small aliquots of the starting amino-oligonucleotides. The S901-Cy5 solid support was deprotected for 24 h at room temperature. Then, the ammonia solutions were removed, and the aqueous solutions were concentrated to dryness. The aptamers and the model sequence (amino-T_8_) were desalted on a NAP-10 column eluted with water. Finally, oligonucleotides were analyzed by HPLC and characterized by MALDI-TOF when possible, as long oligonucleotides (more than 40 nucleotides) gave broad signals (see Supplementary Information Figures S1, S6 and S9A and Table S1).

### 2.2. Conjugation of Aptamers with Hytacs

The solid supports carrying MMT-amino-oligonucleotides were treated first with a solution of 20% piperidine in DMF for 4 min (to remove potential acetyl groups from capping reactions attached to CPG [40]) and were washed with acetonitrile ACN and DCM. Next, the MMT groups were removed, using a solution of 3% TCA in DCM for 5 min, and repeated until no yellow color was observed. The solid supports were washed with DCM, neutralized with 5% diisopropylethylamine (DIPEA) in DCM, and washed again with DCM, ACN, and *N*,*N*-dimethylformamide (DMF).

#### 2.2.1. Conjugation of Aptamers with Adamantyl Groups

A mixture of adamantane-1-carbonyl chloride (20 equiv) and triethylamine (TEA) (40 equiv) in 0.2–0.3 mL of DMF was added to the solid support containing the 5′-amino-oligonucleotides and allowed to react for 4–5 h at 40 °C. The solid supports were then washed with DMF and recoupled with a fresh mixture of adamantane-1-carbonyl chloride (20 equiv) and TEA (40 equiv) in 0.2–0.3 mL of DMF were performed overnight at 40 °C. Finally, the solid support aptamers were washed with DMF and ACN and dried by passing nitrogen over the solid supports. Alternatively, the adamantyl group can be added by a reaction of amino-oligonucleotide supports with Fmoc-Ala-L (adamantyl)-COOH (Iris Biotech). Fmoc-Ala-L (adamantyl)-COOH (20 equiv) were mixed with PyBOP (20 equiv) and DIPEA (40 equiv) in a small volume of DMF and allowed to activate for 10 min. Then, the mixture was added to the solid support and allowed to react overnight at room temperature. After this time, the solid supports were washed with DMF and ACN and dried. Briefly, the solid supports were treated with 1 mL of 32% aqueous ammonia overnight at 65 °C or 24 h at room temperature, (in the case of the S901-Cy5 solid support Hytac-conjugates) and were desalted on a NAP-10 column eluted with water. Finally, oligonucleotide conjugates were analyzed by HPLC and characterized by MALDI-TOF when possible (see Supplementary Information Figures S2, S3, S7, and S9B and Table S1).

#### 2.2.2. Conjugation of Aptamers with Boc_2_-Arg and Boc_3_-Arg

Several samples of Boc_3_-Arg-OH or Fmoc-Arg (Boc)_2_-OH were separately mixed with (20 equiv) of PyBOP and (40 equiv) of DIPEA in a small volume of DMF and allowed to activate for 10 min. Each one of these mixtures was then added to the solid supports, in the smallest possible volume, and allowed to react for 4–5 h at room temperature. After, the solid support aptamers were washed with DMF and a recoupling reaction was carried out overnight. Finally, the solid supports carrying the conjugates were washed with DMF, followed by ACN, and dried by passing air through them. The release of the Hytac-conjugates from the solid support was performed as explained. Briefly, the solid supports were treated with 1 mL of 32% aqueous ammonia overnight at 65 °C or 24 h at room temperature, (in the case of the S901-Cy5 solid support Hytac-conjugates) and were desalted on a NAP-10 column eluted with water. Finally, oligonucleotide conjugates were analyzed by HPLC and characterized by MALDI-TOF when possible (see Supplementary Information Figures S4, S5, S8, and S9C and Table S1).

### 2.3. Generation of Vectors Expressing S1

Different sets of vectors for transient expression of S1 in human cells were prepared. Initially, full-length S and S1 were cloned into the pEGFP-N3 vector. These vectors could express proteins to detectable levels. However, in conjunction with the transfection of the aptamers, expression was not stable enough. Then, a strategy of lentiviral integration of S1 in fusion with a fluorescent protein was carried out. The vector used to generate cell lines was based on the vector V3, expressing S1-Fc-P2A-dTomato, a gift from Dr. Neelamegham (also available as Addgene 164220) [41]. This vector expresses S1 in fusion with Fc, which facilitates solubility of the expressed protein, P2A, a self-cleaving peptide, and the fluorescent protein dTomato. V3 vector is named vector BC527. In order to take advantage of the possible fused proteins expressed from the BC527 vector, by removing regions of the ORFs or by introducing stop codons, vectors expressing FC-dTomato (BC538), S1 (BC534), and S1-FC (BC544), were generated and used in the process of production of cell lines stably expressing S1 in different versions (Figure S10B).

### 2.4. Cell Transfection

Next, 3 × 10^5^ cells were seeded in 6-well plates. After 16 h, they were transfected using Lipofectamine 2000 (11668019, Invitrogen, Carlsbad, CA, USA) with or without aptamers and cultured for 24 h or 48 h. Cells were trypsinized and resuspended in 1 mL of DMEM, to be analyzed by cytometer, or frozen at −80 °C, to successively analyze protein contents by immunoblotting analysis.

### 2.5. Detection of Aptamers and Aptamers-Hytacs

In addition to the detection of our protein of interest, it has been considered essential to make possible the detection of the aptamer-Hytac molecule in cells. Thus, aptamers-Hytacs conjugated with the Cy5 fluorochrome were obtained and used in transfection assays. Then 3 × 10^5^ HeLa cells were seeded in 6-well plates. After 16 h, they were transfected using 8 uL of Lipofectamine 2000 (11668019, Invitrogen) and the indicated aptamer concentration for 48 h. Cells were trypsinized and resuspended in 1 mL of DMEM to be analyzed using a FacsAria I SORP sorter (Beckton Dickinson, San Jose, CA, USA). Aptamers could be detected in cells, confirming the proper internalization. For Cy5 detection, the fluorophore was excited using a red laser (640 nm), and fluorescence was collected by using a 670/14 nm filter.

### 2.6. Generation of Cells Lines Expressing Stable S1

In order to generate cell lines expressing stable S1, HEK293T cells were transfected with the vectors BC527 (S1-FC-dTomato), BC544 (S1-FC), and BC534 (S1) (see Figure S10B), which contain LTR sequences that facilitate the integration of the vector into the host genome along with the vectors necessary to create the viral particles. After 48 h, the medium with the viral particles was collected and transferred to HeLa cells for infection. This process was repeated 72 h post-transfection. HeLa cells were cultured with the medium with the viral particles for 48 h, when the medium was changed. HEK293T cells were also maintained in culture in parallel to HeLa. In this case, no antibiotic selection was carried out since the selection marker Zeocin was toxic at the concentrations previously tested. The cells were observed under the microscope to evaluate the fluorescence levels by dTomato and, after 8 days, single-cell sorting was done with the sorter–fusion cytometry unit. The cells were cultured until they grew enough to obtain a protein extract to perform a western blot. A detailed depiction of the procedures for the generation of the cell lines is included in Figure S10A.

### 2.7. Flow Cytometry

The fluorescence was determined using a FacsAria I SORP sorter (Beckton Dickinson) by counting 10,000 events. Excitation of the sample was done using a blue (488 nm) laser for the forward scatter (FSC) parameter. A green-orange laser (561 nm) was used to collect the side scatter (SSC) signal, and a UV laser (350 nm) was used for DAPI excitation. Cells were gated according to their scatter (FSC vs. SSC) parameters; doublets were discriminated using their FSC-h signal. For dTomato detection, excitation and emission wavelengths were 554 nm and 583 nm, respectively. Cy5 was excited using a red (640 nm) laser and a 670/14 nm filter was used to collect fluorescence. For cell viability analysis, DAPI was added to cells just before cytometer analysis at a final concentration of 0.1 µg/mL. A schematic representation of the cytometric analysis, including cell viability assessment, is shown in Figure S11.

### 2.8. Immunoblotting Analysis

Cell extracts were separated by electrophoresis on 10% acrylamide gels and transferred onto PVDF membranes. After blocking the membranes with 5% non-fat milk in T-TBS, they were cut along the 43 kDa marker line and incubated for 16 h with anti-S1 antibody 1:1000 in T-TBS (ab283942, Abcam, Cambridge, UK) or anti-Spike RBD antibody 1:1000 in T-TBS (40592-T62, Abyntek Biopharma, Derio, Spain) and the anti-GAPDH antibody 1:1000 in T-TBS (sc-32233, Santa Cruz Biotechnology, Sant Cruz, CA, USA), and for one hour with anti-rabbit antibody 1:10,000 in T-TBS (NA934V, Merck, Darmstadt, Germany) for anti-S1 and anti-Spike RBD antibodies and anti-mouse 1:10,000 in T -TBS (32430, Invitrogen) for anti-GAPDH antibody. The membrane was developed using ECL prime (12172708346, Amersham Biosciences Corporation, Arlington Heights, IL, USA) and films (47410, Fujifilm Co., Tokyo, Japan).

### 2.9. Degradation Assays Using Aptamer-Hytacs

Having obtained different lines with stable expression of Spike S1, transfection experiments of the aptamers were carried out to degrade the Spike S1 protein. To do this, 3 × 10^5^ HeLa BC527 and HEK293T BC527 cells (Spike S1-FC; dTomato) or HEK293T BC544 cells (Spike S1) were seeded in 6-well plates. After 16 h, they were transfected using 8 μL of Lipofectamine 2000 (11668030, Thermo Fisher Scientific, Waltham, MA, USA) and the indicated concentration of aptamers-Hytacs and cultured for 24 or 48 h. Cells were then trypsinized and resuspended in 1 mL of DMEM to be analyzed using a FacsAria I SORP sorter (Beckton Dickinson). Finally, the cells were frozen at −80 °C, lysed in lysis buffer (50 mM Tris-HCL pH 7.4, 100 mM NaCl, 1% Triton free Protease Inhibitor Cocktail Tablets) and the protein amount was normalized by Bradford to be analyzed by immunoblotting analysis. Figure 4 includes a graphical representation of assay replicates and unpaired *t*-test analysis (GraphPad Prism 10.3.0, GraphPad Software, San Diego, CA, USA).

## 3. Results

### 3.1. Conjugation of Aptamers to Hytacs

Due to their validation in the literature, we selected adamantane [12] and Boc_3_-Arg [13] as hydrophobic tags to incorporate into the S901 and S702 aptamers, exhibiting affinity to the Spike S1 domain of SARS-CoV-2 (the sequence of aptamers and the process followed to obtain them by SELEX is not included in this work). We decided to include a reactive aminoalkyl group at the 5′-position in order to direct the incorporation of hydrophobic tags to this position as the 3′-position will be used for the addition of biotin or Cy5 fluorescent tags. The optimized versions of the aptamers were conjugated to the Hytacs by formation of an amide bond between the 5′-amino-end of the different aptamers and the Hytac-carrying carboxylic group derivatives, while the assembled oligonucleotide was still attached to the solid support. The incorporation of an alkylamino group at the 5′end was done using the MMT-6-aminohexyl phosphoramidite. As the aptamers are relatively long oligonucleotides and difficult to characterize by mass spectrometry, we decided to use a short oligonucleotide sequence (5′-aminohexyl-T_8_) to set up the optimal conditions for the preparation of Hytac conjugates (see Supplementary Information).

**Figure 1:**
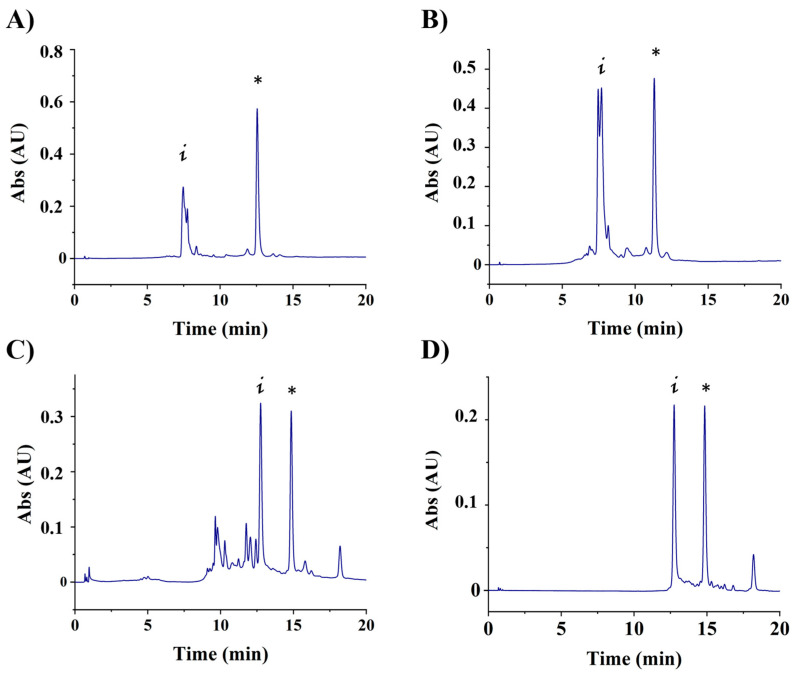
HPLC profiles of the adamantyl conjugates. (**A**) S901, (**B**) S702, (**C**) S901-Cy5 at 260 nm and (**D**) S901-Cy5 at 550 nm. * Indicates the position of the desired conjugates, “i” indicates the position of the initial amino-oligonucleotide.

Oligonucleotide–adamantane conjugates have been previously prepared in order to form host–guest complexes with beta-cyclodextrin derivatives to increase cellular uptake [42,43,44,45]. In this work, we reacted 5′-amino-oligonucleotides with an excess of the adamantane-1-carbonyl chloride in the presence of triethylamine or with Fmoc-L-Ala (adamantyl)-COOH with PyBOP. In both cases, the reaction was found to be slow due to the steric hindrance of the adamantyl group, but the best results were obtained with a double coupling protocol using adamantane-1-carbonyl chloride obtaining the desired compound as the major compound (Figures S2, S3 and S7, and Figure 1). Adamantyl-conjugates were characterized by mass spectrometry when possible (Table S1).

**Figure 2:**
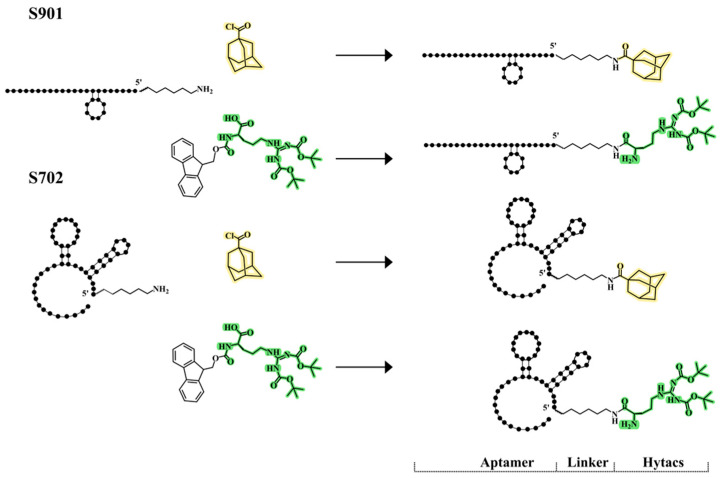
Schematic representation of the chimeras generated in this work. Aptamers S901 and S702, represented with their linker functionalized with an amine end (**left**) at the 5′ end, were conjugated to adamantane (highlighted in yellow) and tert-butyl groups (highlighted in green), to generate final constructs (**right**).

**Figure 3:**
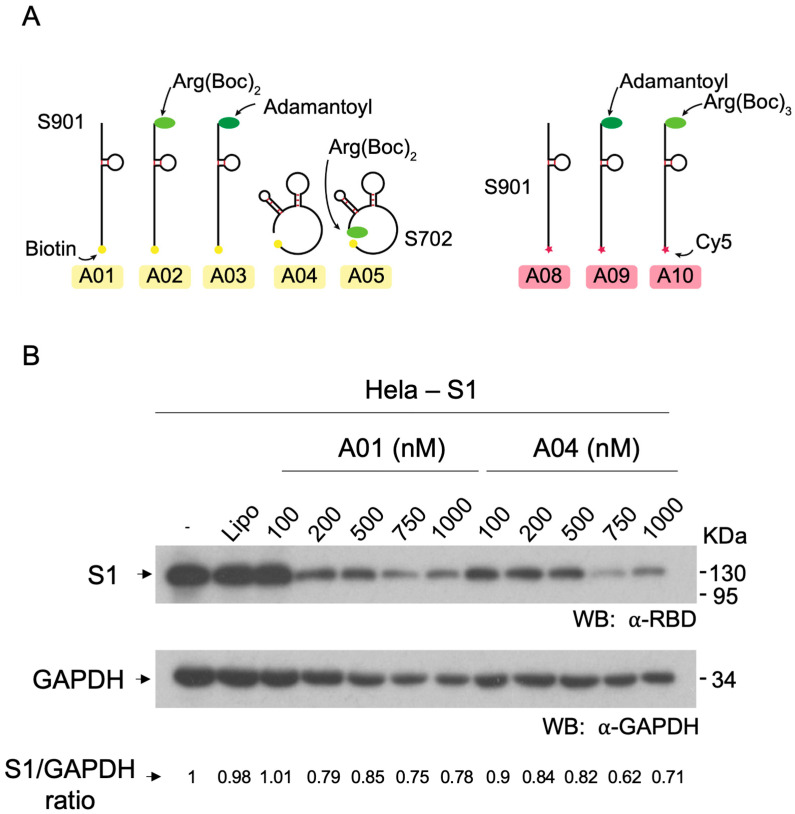
Characterization of S1-targeting aptamers. (**A**) Scheme showing the aptamers and aptamer-Hytacs used in this work. (**B**) Effect of increasing concentrations of S901 (**A01**) and S702 (**A04**) aptamers on S1 labels.

The reaction between solid-phase-supported amino-oligonucleotides and Boc-Arg (Boc)_2_-COOH or Fmoc-Arg (Boc)_2_-COOH was performed by activation of the carboxylic functions either with HATU or PyBOP in an anhydrous organic solvent. Both reagents were efficient in the synthesis of T_8_-conjugates, but the use of PyBOP gave better results (Figures S4 and S5). When using Boc-Arg (Boc)_2_-COOH, we obtained the conjugate with three Boc groups (Arg-Boc_3_) but when using Fmoc-Arg (Boc)_2_-COOH we obtained the conjugate with two Boc groups (Arg-Boc_2_) as the Fmoc group is labile to ammonia deprotection. For the preparation of the aptamer conjugates, we chose the use of PyBOP to obtain the expected conjugates as determined by phase HPLC (Figure S8) analysis. The identities of the purified conjugates were confirmed by MALDI-TOF when possible (Table S1). A schematic representation of the generated chimeras is shown in Figure 2 and Figure 3A,B.

### 3.2. Cell Lines Stably Expressing S1

To generate human cells stably expressing S1, HEK293T cells were transfected with dTomato vectors expressing S1 and controls (BC538, BC527, BC534, and BC544; see Figure S10B). These vectors contain LTR sequences that facilitate the integration of the vector into the host genome. Along with S1 expression vectors, vectors promoting the formation of lentiviral particles were co-transfected. After 48 h, the medium with the viral particles was collected and transferred to HeLa cells for infection. Cells were observed under the microscope to evaluate the fluorescence levels by dTomato and after 8 days single cell sorting was performed. The whole process is described in the methods and in Figure S10A, and the yields and clones generated are shown in Figure S10C. The final selected cell lines were validated by Western blot. The HeLa-S1 lines and HEK293-S1, expressing S1-dTomato and other constructs generated (see Figure S10C,D) were stocked for further experiments.

### 3.3. Efficiency of Aptamer Delivery to Human Cells

**Figure 4:**
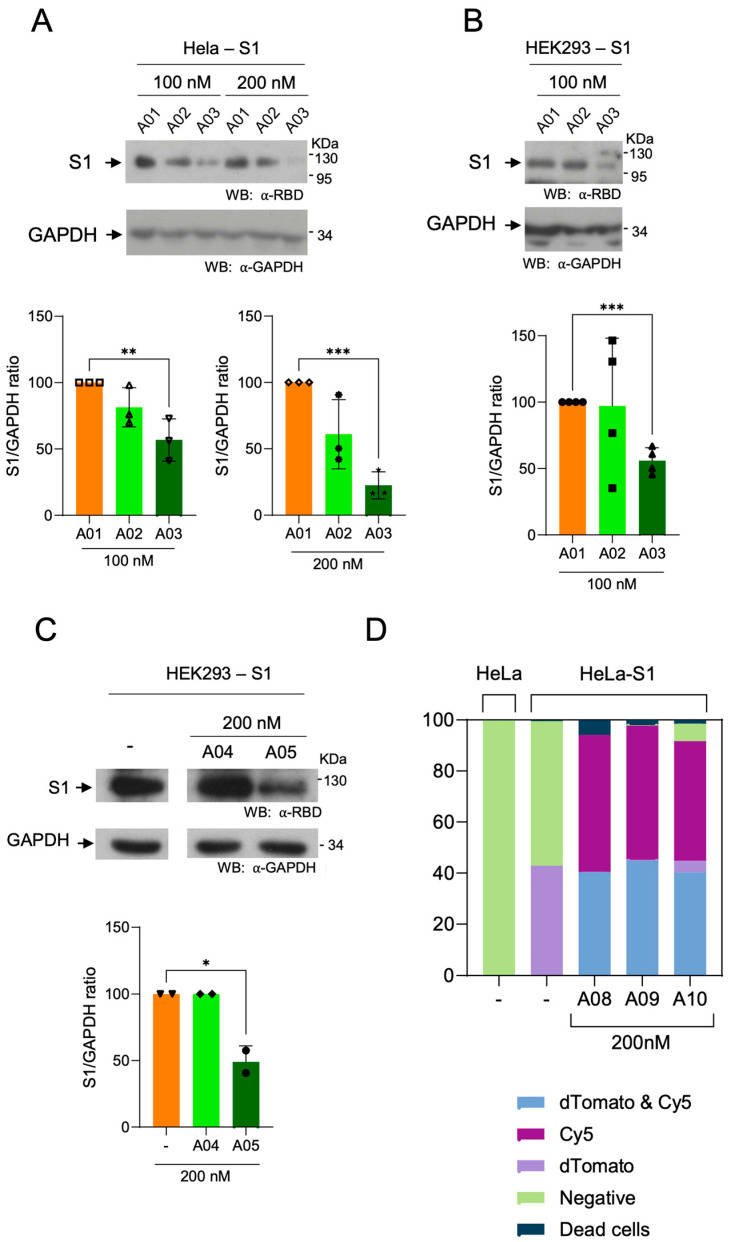
Degradation of S1 in the presence aptamer-Hytacs. (**A**) Treatments of HeLa cells expressing S1 (HeLa-S1) with **A01**, **A03,** and **A03** at two different concentrations (100 and 200 nM). (**B**) Treatments of HEK393 expressing S1 (clone BC527) with **A01**, **A03**, and **A03** at 100 nM. (**C**) Treatments of HEK393 cells expressing S1 (clone BC544) with **A04** and **A05** at 200 nM. Asterisks indicate statistical significance at * *p* ≤ 0.05; ** *p* ≤ 0.01; *** *p* ≤ 0.001 (unpaired, two-tail *t*-test). (**D**) Flow cytometry quantification efficiency of transformation of Cy5-aptamers in HeLa cells expressing S1-dTomato (clone BC527).

In order to ensure that aptamers were efficiently delivered to cells upon transfection, a set of aptamers carrying the Cy5 fluorophore was prepared and tested (compounds **A08**, **A09**, and **A10**; Figure 3A, right panel). In these assays, 200 nM Cy5-labeled aptamers were transfected in HeLa cells and Cy5 fluorescence was determined by flow cytometry. It was observed that aptamers internalized correctly, with more than 85% internalized signal (see Figure 4D), exhibiting a high dwelling time in cells. Moreover, low toxicity was observed in cell viability assays and in evaluation of alive/dead cells proportion (Figures S11 and S12).

### 3.4. Effect of Hytac-Free Aptamers on S1 Levels

In order to evaluate the effect of aptamers **A01** and **A04** on the levels of S1, a dose-response assay was carried out. Treatments of aptamers at 100, 200, 500, 750, and 1000 nM were carried out for 48 h in HeLa cell line stably expressing S1 (BC527). It was observed that untreated cells and cells treated with vehicle (lipofectamine), exhibited the same levels of S1 (Figure 3B). Moreover, treatments with 100 nM of aptamer **A01** did not exert changes in S1 levels. However, concentrations from 200 to 1000 nM of aptamer **A01** caused a decrease in S1. Treatments with aptamer **A04** caused decreases in S1 levels, showing a slight decrease at 100, 200, and 500 nm, and a strong decrease at 750 and 1000 nM. These observations suggest that unconjugated aptamers have the capacity to interfere with the steady-state levels of S1 at high concentrations. In order to determine the effect of Hytac conjugates on S1 levels, further experiments were carried out using aptamers at 100–200 nM, including the corresponding unconjugated controls.

### 3.5. Degradation of Spike S1 by Aptamers-Hytacs

Degradation assays were performed in Hela-S1 and HEK293-S1 cells (See Figure S10), in order to validate results in distinct cellular types. Unconjugated aptamer S901 (**A01**), and aptamers conjugated to Arg-Boc_2_ (**A02**) and adamantyl (**A03**) were tested at 100 nM (Figure 4A, left lanes; and Figure 4B). A strong decrease of S1 levels in cells treated with **A03** and a moderate decrease in cells treated with **A02** were observed. Moreover, Hela-S1 cells were treated with aptamers at 200 nM. Despite a slight instability observed in the S1 signal in treatments with aptamer **A01** at 200 nM (Figure 3B, lane 4), a more prominent decrease was observed when **A02** and **A03** were used (Figure 4A, lanes 5 and 6). Similarly, treatment of cells with compound **A05** (aptamer S702 modified with Arg-Boc_2_) at 200 nM induced a decrease in S1 levels in HEK293 cells, as compared to the unmodified aptamer, **A04** (Figure 4C). The permeability of S901 aptamer (**A08**) and the derivate chimeras containing adamantyl (**A09**) and Arg-Boc_3_ (**A10**) was evaluated in parallel. As observed previously (Figures S11 and S12), these compounds exhibited high permeability and low toxicity at 200 nM (Figure 4D).

## 4. Discussion

Targeted protein degradation opens the possibility of designing chimeras that induce the elimination of critical protein factors in pathological and infectious processes. The huge flexibility of this approach has combined multiple ligands of proteolysis effectors with a variety of linkers and ligands of protein targets. Among the repertoire of types of molecules recognizing and binding targets, oligonucleotide aptamers appear as a group with great potential due to several reasons. One of them is that the methodology to develop aptamers as high-affinity ligands to targets is highly straight forward and feasible. In addition, Selex can be applied towards structurally and mechanistically uncharacterized targets, which opens the possibility of generating ligands for completely novel targets. Furthermore, as a general trait, aptamers are permeable, stable, and can be produced with reduced costs [28,31].

Despite the high profile of aptamers as tools in biomedicine, little is known about the efficacy and versatility of aptamers acting as degraders. Until now, very few examples of aptamers linked to degradation ligands have been reported (mentioned in the introduction), and, to our knowledge, there are no examples of aptamers linked to hydrophobicity tags in the literature. In the present work, we present the first aptamers conjugated to adamantane and to tert-butyl groups. These new sets of compounds include aptamers S702 and S901 that bind to S1, generated by the Selex method (the procedure of production and the sequence of these aptamers is unpublished). These novel types of aptamers-based degrading chimeras have been tested in human cells expressing the SARS-CoV-2 factor S1. Aptamers have exhibited permeability and capacity to downregulate S1 in two distinct human cell types. Moreover, aptamers were likely not trapped by endosomes, since degradation effects were observed within the 24–48-h range, and no fluorescence quenching effect [46,47] was observed in the transfections using Cy5-labeled aptamers analyzed by cytometry throughout the study. With these observations, hydrophobic tags appear as robust degradation ligands also linked to aptamers. Further research will be required to determine the applicability of aptamer-Hytacs in biomedicine.

## 5. Conclusions

Chimeras containing oligonucleotide aptamers linked to hydrophobic tags have been produced and tested. In the present conceptualization, we conjugated aptamers exhibiting high affinity towards the S1 domain of SARS-CoV-2 spike protein to adamantane and tert-butyl-carbamate moieties. We observed that these compounds could be efficiently internalized into cultured cells. Moreover, it was consistently observed that the S1 cellular levels could be reduced by treating cells with chimeras containing an adamantyl group. These observations suggest that aptamer-Hytacs could behave as proteolytic chimeras against intracellular protein targets, and highlight the potential of aptamer-based strategies in drug development.

## Data Availability

All data are available upon request to corresponding authors.

## Supplementary Materials

The following supporting information can be downloaded at: https://www.mdpi.com/article/10.3390/cells13211767/s1, Figure S1: Characterization of the starting NH2-T8 oligonucleotide. Figure S2: Characterization of the model sequence conjugated to adamantyl. Figure S3: Characterization of the Ala-L-adamantyl-NH-T8 oligonucleotide conjugated. Figure S4: Characterization of the Boc2-Arg-NH-T8 oligonucleotide conjugate. Figure S5: Characterization of the Boc3-Arg-NH-T8 oligonucleotide conjugate. Figure S6: HPLC profiles of the different aptamers. Figure S7: HPLC profiles of the Adamantyl-aptamers conjugates. Figure S8: HPLC profiles of the Boc2-Arg- and Boc3-Arg- aptamers conjugates. Figure S9: MALDI-TOFF characterized of the S901-Cy5 and the Hytacs conjugates. Figure S10: Generation of Cell lines expressing stable S1. Figure S11: Cell viability of cells transfected with aptamers. Figure S12: Microscopy images of cells treated with aptamer-Hytacs. Table S1: MALDI-TOFF characterization
