# Supplementary material for: Aptamer-Hytac Chimeras for Targeted Degradation of SARS-CoV-2 Spike-1
Source: Cells. 2024 Oct 25;13(21):1767. doi: 10.3390/cells13211767 (PMC11544835; doi:10.3390/cells13211767)
Supplement: Supplementary file 1 [file cells-13-01767-s001.zip › cells-3211466-supplementary.pdf]

Supplementary information

Aptamer-Hytac chimeras for targeted degradation of SARS-CoV-2 Spike-1

Carme Fàbrega [1,2,†], Núria Gallisà-Suñé [3,†], Alice Zuin [3], Juan Sebastián Ruíz [4], Bernat Coll-Martínez [3], Gemma Fabriàs [5], Ramon Eritja [1,2,*] and Bernat Crosas [3,*]

1. Materials and Methods.
2. Conjugation of the Hytacs with the model sequence (NH_2-T_8).
    2.1. Conjugation protocol using adamantane-1-carbonyl chloride.
        2.1.1. Conjugation in one step.
        2.1.2 Conjugation using a recoupling step.
    2.2. Conjugation protocol using PyBOP as activating agent.
    2.3. Conjugation protocol using HATU as activating agent.
3. Release and deprotection of the Hytacs model sequence conjugates.
4. HPLC profiles and MALDI-TOF analysis of the control model sequence and the Hytacs.
    Figure S1: Characterization of the starting NH_2-T_8 oligonucleotide.
    Figure S2: Characterization of the model sequence conjugated to adamantyl.
    Figure S3: Characterization of the Ala-L-adamantyl-NH-T_8 oligonucleotide conjugated.
    Figure S4: Characterization of the Boc_2-Arg-NH-T_8 oligonucleotide conjugate.
    Figure S5: Characterization of the Boc_3-Arg-NH-T_8 oligonucleotide conjugate.
5. HPLC profiles of the different aptamers and their Hytacs conjugates.
    Figure S6: HPLC profiles of the different aptamers.
    Figure S7: HPLC profiles of the Adamantyl-aptamers conjugates.
    Figure S8: HPLC profiles of the Boc_2-Arg- and Boc_3-Arg- aptamers conjugates.
6. MALDI-TOFF
    Figure S9: MALDI-TOFF characterized of the S901-Cy5 and the Hytacs conjugates.
7. Cellular assays with aptamer-Hytacs
    Figure S10. Generation of Cell lines expressing stable S1.
    Figure S11. Cell viability of cells transfected with aptamers
    Figure S12. Microscopy images of cells treated with aptamer-Hytacs.

Table S1. MALDI-TOFF characterization

1. Materials and Methods

Reagents and solvents were purchased at the highest commercial quality available. All chemical reactions were carried using anhydrous solvents. Ultrapure water (Millipore) was used in all experiments. Adamantane-1-carbonyl chloride, Fmoc-Arg(Boc)$_2$-OH and Boc$_3$-Arg-OH were obtained from commercial sources Alfa Aesar, Novabiochem and Abcr GmbHcat respectively. Fmoc-L-Ala-(adamantyl)-COOH was obtained from Iris Biotech.

The aptamers S901 and S702 carrying biotin at the 3'end used in ELONA experiments were purchased from Biomers and used directly as received. The synthesis of Hytacs conjugates was performed with oligonucleotides carrying an alkylamino group at the 5'-position. A short oligonucleotide sequence (5'-aminohexyl-T8) was prepared in our laboratory using monomethoxytrityl (MMT)-6-aminohexyl phosphoramidite for the incorporation of an alkylamino group at the 5'end. This oligonucleotide was used to set up the optimal conditions for the preparation of Hytacs conjugates. Then, 5'-aminohexyl-aptamers S901 and S702 with or without biotin at the 3'-end were purchased to Biomers on a 1 µmol scale. In addition, the two aptamers contain at the 3' end a Biotin-TEG followed by a Spacer C18. 5'-Aminohexyl-S901-3'-Cy5 oligonucleotide was synthesized in our laboratory on a 1 µmol scale with standard phosphoramidites and ancillary reagents obtained from Applied Biosystems and LGC Link Technologies, on a H-8 DNA synthesizer (K&A Laboratories) at 1-µmol scale. The solid support functionalized with Cyanine 5 and the N-MMT-6-aminohexyl phosphoramidite were purchased to Glen research and Biosearch technologies respectively. Analytical RP-HPLC was performed using an XBridge OST C18 2.5 µm column and a Nucleosil Analytic column 120 C18 (250x4mm). Semipreparative RP-HPLC was performed on a Waters chromatography system with a 2695 Separations Module equipped with a Waters 2998 Photodiode Array Detector using Nucleosil 120 C18 (250x8mm) column. Solvents for HPLC were prepared using triethylammonium acetate (TEAAc) and acetonitrile as mobile phase. Buffer A: 5% ACN in 0.1 M TEAAc buffer in H_2O. Buffer B: 70% ACN in 0.1 M TEAAc. DNA purification cartridges were from Glen Research. The sephadex-G25 (NAP-10) column were from GE Healthcare. Molecular absorption spectra from 220 to 320 nm were recorded with Hellman quartz cuvettes, in a Jasco V650 spectrophotometer. Mass spectra were obtained with a MALDI Voyager DETM RP time-of-flight (TOF) spectrometer using 2',4',6'-trihydroxyacetophenone monohydrate (THAP, Aldrich) and ammonium citrate dibasic (Fluka).

2. Conjugation of the Hytacs with the model sequence (NH$_2$-T$_8$).

The MMT-aminohexyl-T$_8$ oligonucleotide was treated with a solution of 20% piperidine in DMF for 4 min to remove potentially reactive acetyl groups. The solid support was then washed with DMF, ACN and DCM. The 5'-MMT group was removed using a solution of 3%

TCA in DCM for 5 min. This process was repeated several times until no yellow color was observed. Next, the solid support was washed with DCM and neutralized with 5% DIPEA in DCM and washed again with DCM, CH_3CN and DMF.

A small amount of the 5'-aminohexyl-T_8 oligonucleotide solid support was directly deprotected with 32% aqueous ammonia during 2 h at room temperature, analyzed by HPLC and characterized by MALDI-TOF as control sequence (NH_2-T_8, Figure S1).

2.1. Conjugation protocol using adamantane-1-carbonyl chloride.

2.1.1. Conjugation in one step.

A mixture of Adamantane-1-carbonyl chloride (20 equiv.) and TEA (40 equiv.) in 0.2-0.3 ml of DMF is added to the solid support with the NH_2-T_8 and left react overnight at 40 °C. Then, the solid support is washed with DMF and ACN and dried by-passing air or nitrogen. Alternatively, the adamantyl group can be added by reaction of amino-oligonucleotide supports with Fmoc-Ala-L(adamantyl)-COOH (Iris Biotech). Fmoc-Ala-L(adamantyl)-COOH (20 equiv) were mixed with PyBOP (20 equiv) and DIPEA (40 equiv) in a small volume of DMF and allowed to activate for 10 min. Then, the mixture was added to the sold support and allowed to react overnight at room temperature. After this time, the solid supports were washed with DMF and ACN and dried.

2.1.2 Conjugation using a recoupling step.

It is similar to the protocol in 2.1.1, with the addition of a recoupling with fresh Adamantane-1-carbonyl chloride and TEA. Briefly, a mixture of Adamantane-1-carbonyl chloride (20 equiv.) and TEA (40 equiv.) in 0.2-0.3 ml of DMF is added to the solid support with the NH_2-T_8 and left 2-5 h at 40 °C. After this time, the support is washed with DMF and fresh Adamantane-1-carbonyl chloride (20 equiv.) and TEA (40 equiv.) in 0.2-0.3 ml of DMF were added to the solid support to performed a re-coupling overnight at 40 °C. Finally, the solid support is washed with DMF and ACN and dried by-passing air or nitrogen over the solid support.

2.2. Conjugation protocol using PyBOP as activating agent.

The Boc_3-Arg-OH and Fmoc-Arg$(Boc)_2$-OH were separately mixed with (20 equiv.) of PyBOP and (40 equiv.) of DIPEA in a small volume of DMF and allowed to activate for 10 min more or less. Each one of these mixtures was then added to 7 mg of the NH_2-T_8 oligonucleotide

solid support (in the smallest volume possible) and allowed to react for 4-5 h at room temperature. After, the NH_2-T_8 oligonucleotide solid supports were washed with DMF and a re-coupling was carried out overnight. Finally, the NH_2-T_8 oligonucleotide solid supports were washed with DMF followed with ACN and dried by passing air through it.

2.3. Conjugation protocol using HATU as activating agent.

The Boc_3-Arg-OH and Fmoc-Arg$(Boc)_2$-OH (20 equiv.) were mixed for 10 min with HATU (20 equiv.) and DIPEA (40 equiv.) in 0.2-0.3 ml. Then, this mixture was added to the solid support and left to react for 5 h at room temperature. After, the solid support was washed with DMF and a re-coupling was performed overnight. Finally, the solid support was washed with DMF and ACN and it is dry by-passing air or nitrogen over the solid support.

3. Release and deprotection of the Hytacs model sequence conjugates.

The Hytac-NH-T_8 oligonucleotide solid support conjugates were transferred to vials with screw caps and 1 ml of 32% aqueous ammonia was added and allowed to react for 1 h at room temperature. The solutions were filtered to separate the Hytacs-NH-T_8 oligonucleotide conjugates from the solid supports. Then, the ammonia solutions were removed by passing N_2 through them and the aqueous solutions were concentrated to dryness. The resulting Hytacs-NH-T_8 oligonucleotide conjugates were desalted on a NAP-10 column eluted with water. Finally, the Hytacs-NH-T_8 oligonucleotide conjugates were analyzed by HPLC, (Figures S2A, S3A, S4A, S4B, S5A and S5B) with a gradient of 0-75% buffer B in 30 min, and characterized by MALDI-TOF (Figure S2C, S3B, S4C and S5C).

4. HPLC profiles and MALDI-TOF analysis of the NH_2-T_8 oligonucleotide model sequence and its Hytacs conjugates.

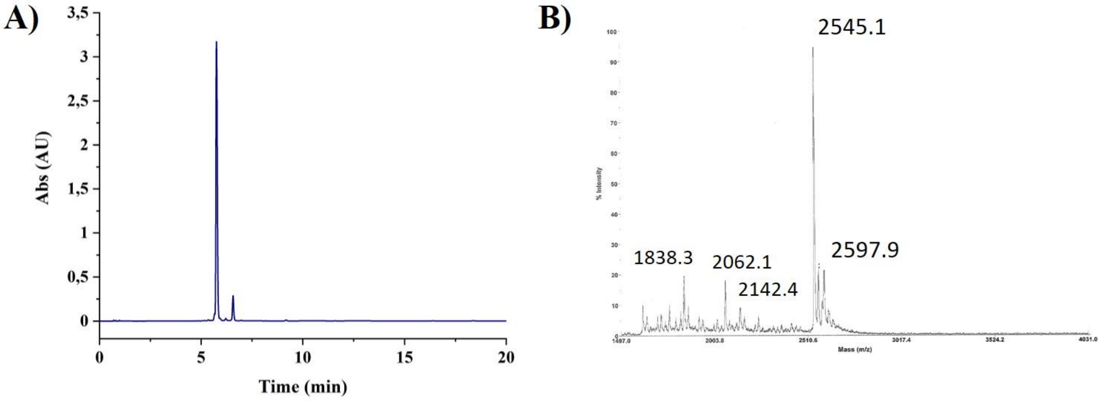

Figure S1: Characterization of the starting NH_2-T_8 oligonucleotide. A) HPLC profile and B) MALDI-TOFF analysis.

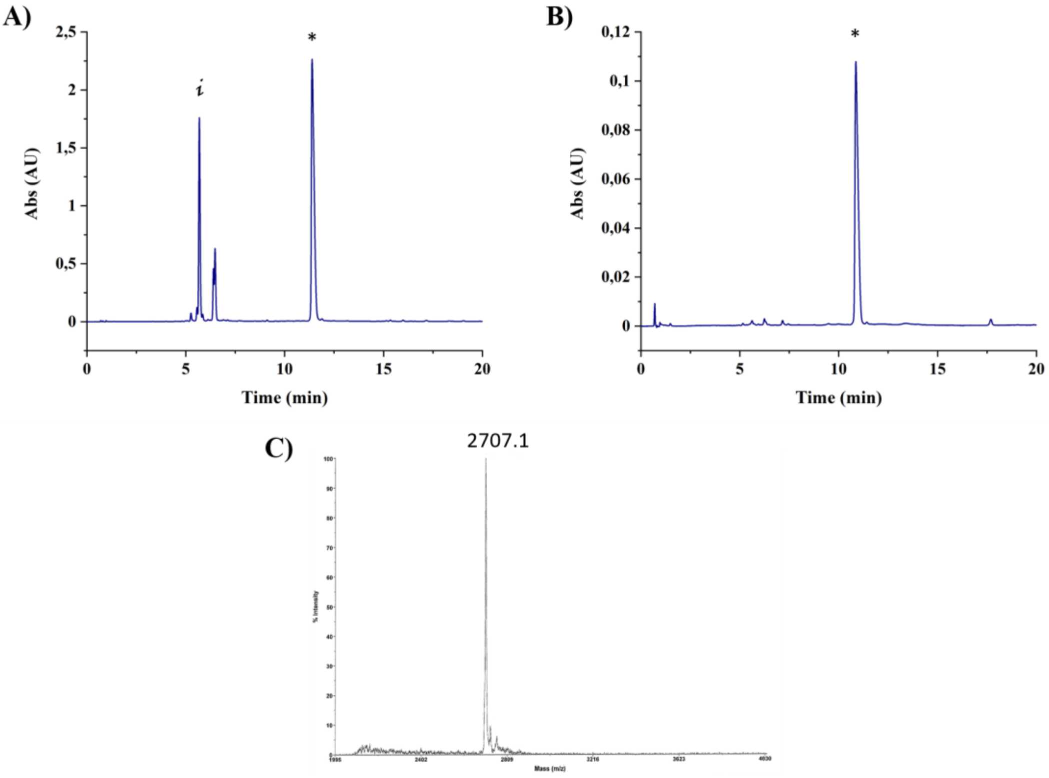

Figure S2: Characterization of the model sequence conjugated to adamantyl (Ada-NH-T_8) using adamantane-1-carbonyl chloride. A) HPLC profiles for the single step reaction at 40 °C for 5 h, B) HPLC profiles after a re-coupling step both at 40 °C and C) MALDI-TOFF analysis. ι correspond to the NH_2-T_8 sequence and * is the Hytac-oligonucleotide conjugated.

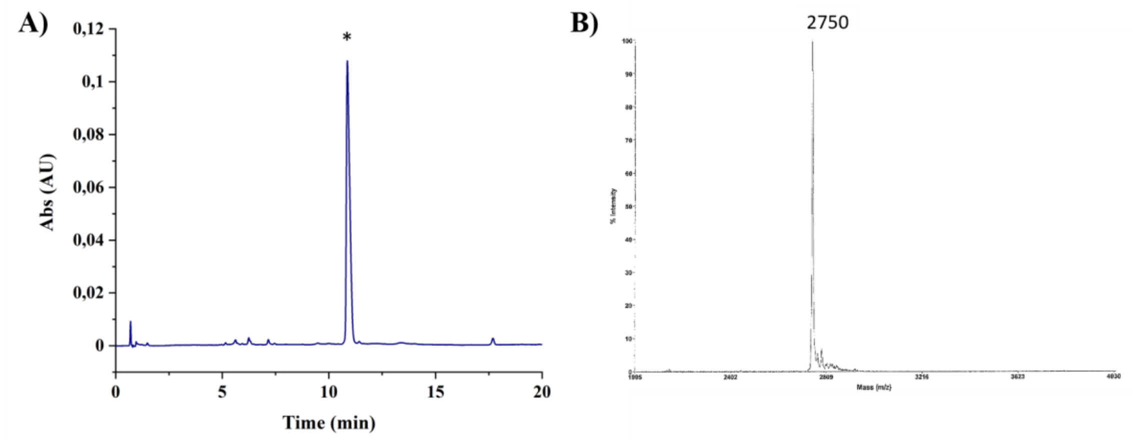

Figure S3: Characterization of the Ala-L-adamantyl-NH-T_8 oligonucleotide conjugated. A) HPLC profile and B) MALDI-TOFF analysis. * is the Hytac-oligonucleotide conjugated.

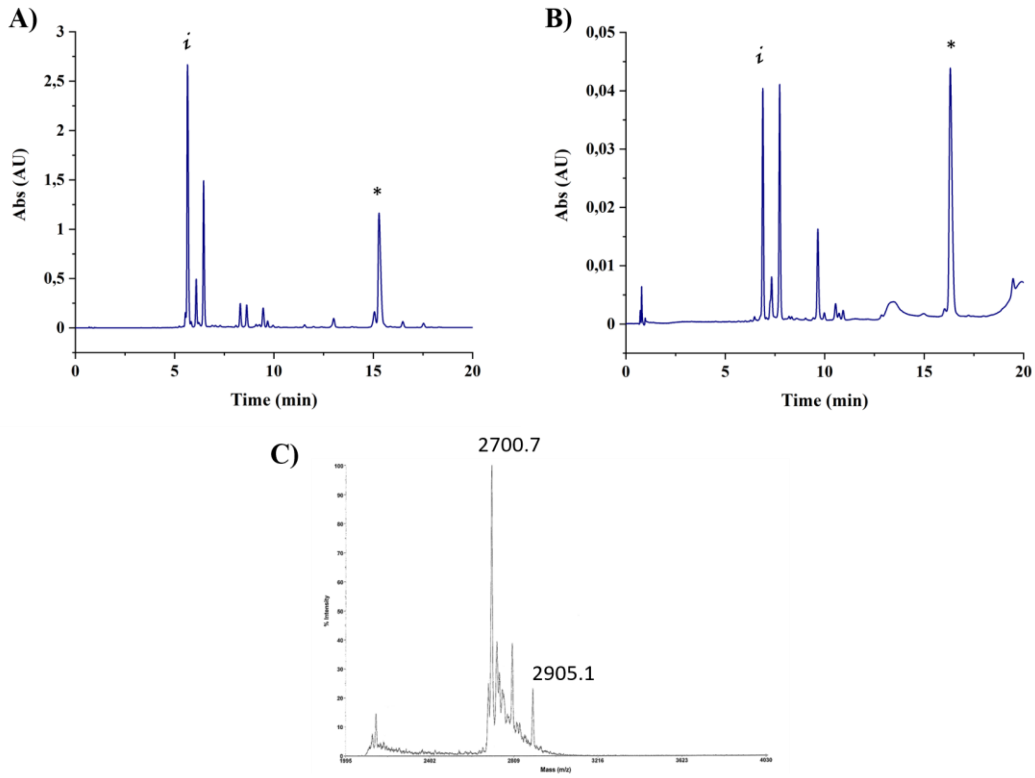

Figure S4: Characterization of the Boc$_2$-Arg-NH-T_8 oligonucleotide conjugate. HPLC profiles using as coupling reagent A) HATU and B) PyBOP. C) MALDI-TOFF analysis. ί corresponds to the NH_2-T_8 sequence and * to the Hytac-oligonucleotide conjugated.

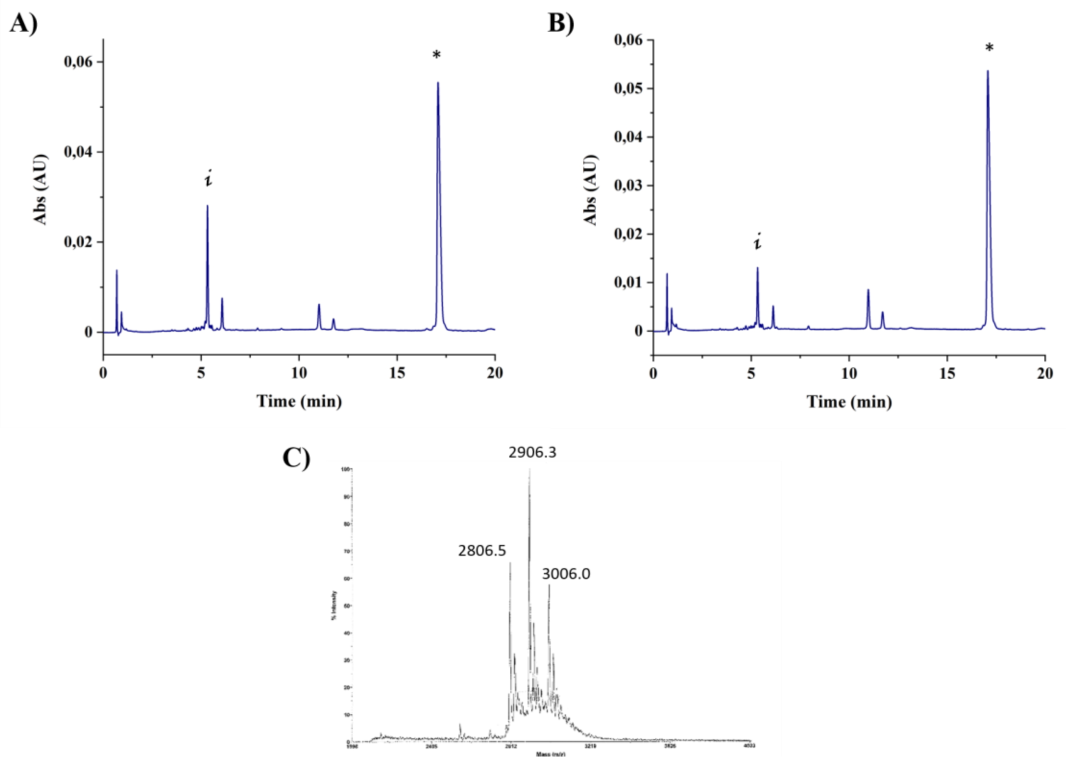

Figure S5: Characterization of the Boc_3-Arg-NH-T_8 oligonucleotide conjugate. A and B) HPLC profiles using as coupling reagent A) PyBOP and B) HATU. C) MALDI-TOFF analysis. ί corresponds to the NH_2-T_8 sequence and * to the Hytac-oligonucleotide conjugated.

5. HPLC profiles of the different aptamers and their Hytacs conjugates.

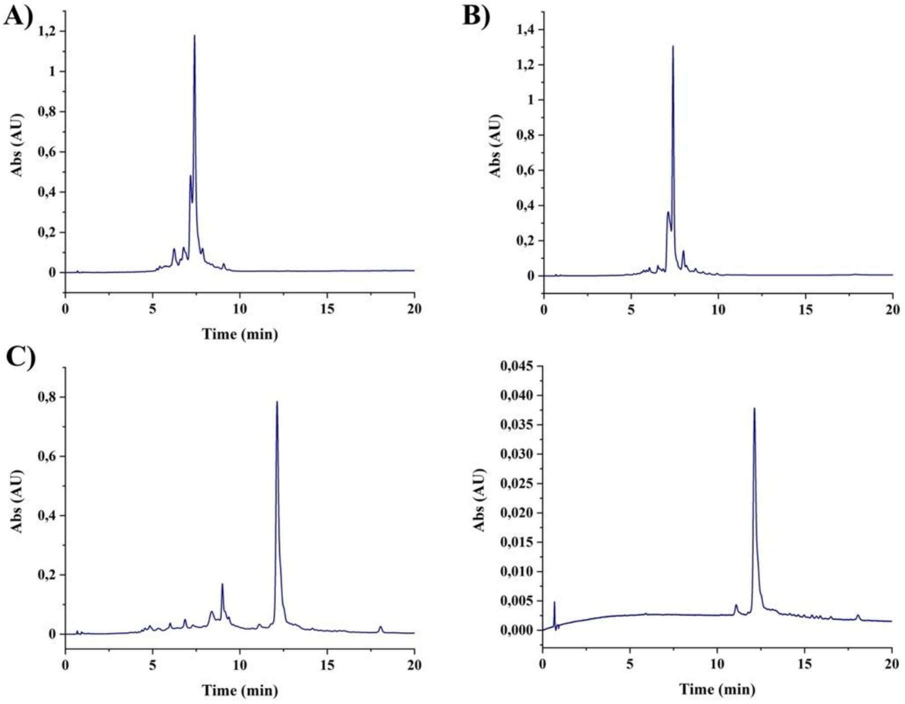

Figure S6: HPLC profiles of the different aptamers. A) S702, B) S901, C) S901-Cy5 at 260 nm and D) S901-Cy5 at 550 nm.

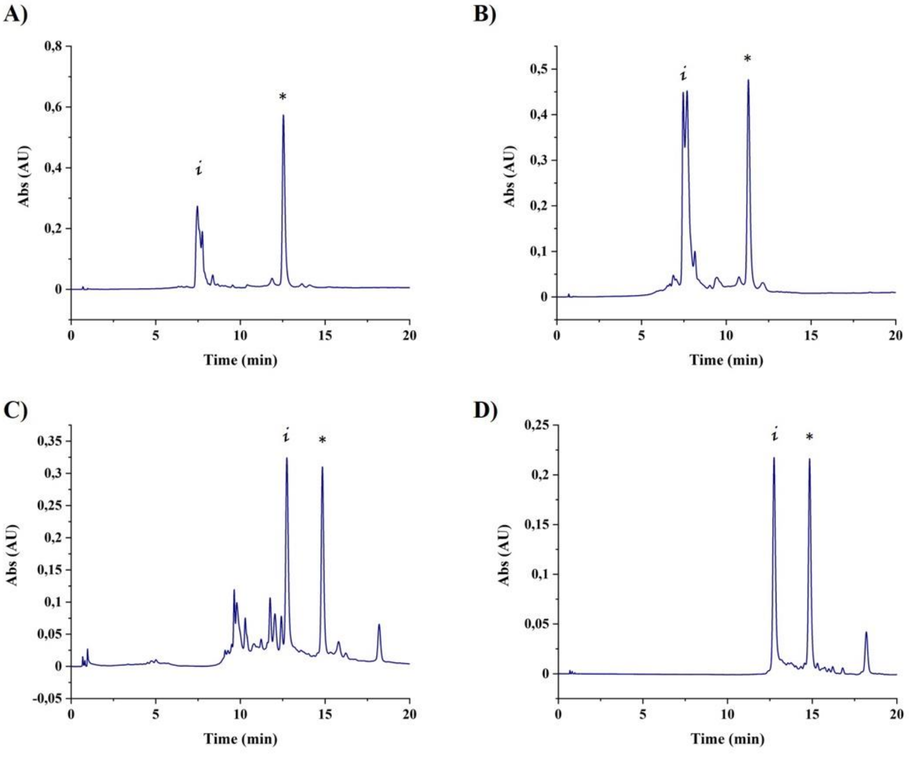

Figure S7: HPLC profiles of the Adamantyl-aptamers conjugates. A) S901, B) S702, C) S901-Cy5 at 260 nm and D) S901-Cy5 at 550 nm. * Corresponds the Hytac-oligonucleotide conjugated.

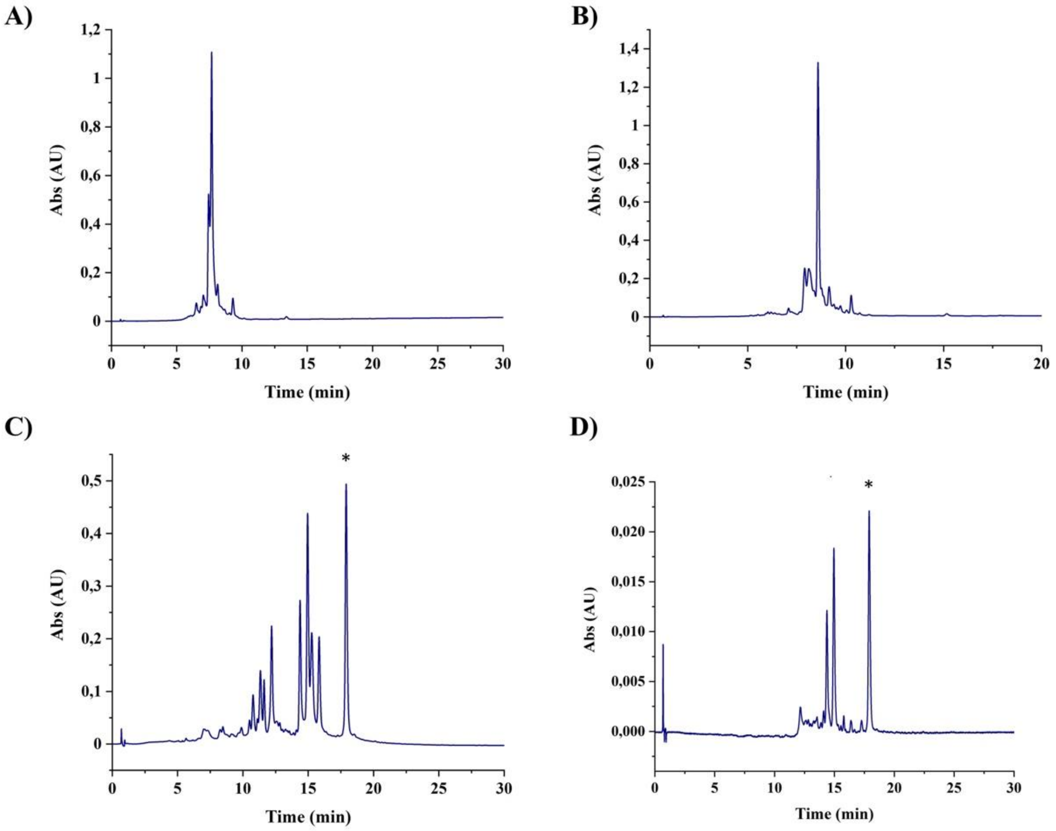

Figure S8: HPLC profiles of the Boc$_2$-Arg- (A and B) and Boc$_3$-Arg- (C and D) aptamers conjugates. A) S702, B) S901 C) S901-Cy5 at 260 nm and D) S901-Cy5 at 550 nm. * Corresponds to th Hytac-oligonucleotide conjugated.

6. MALDI-TOFF

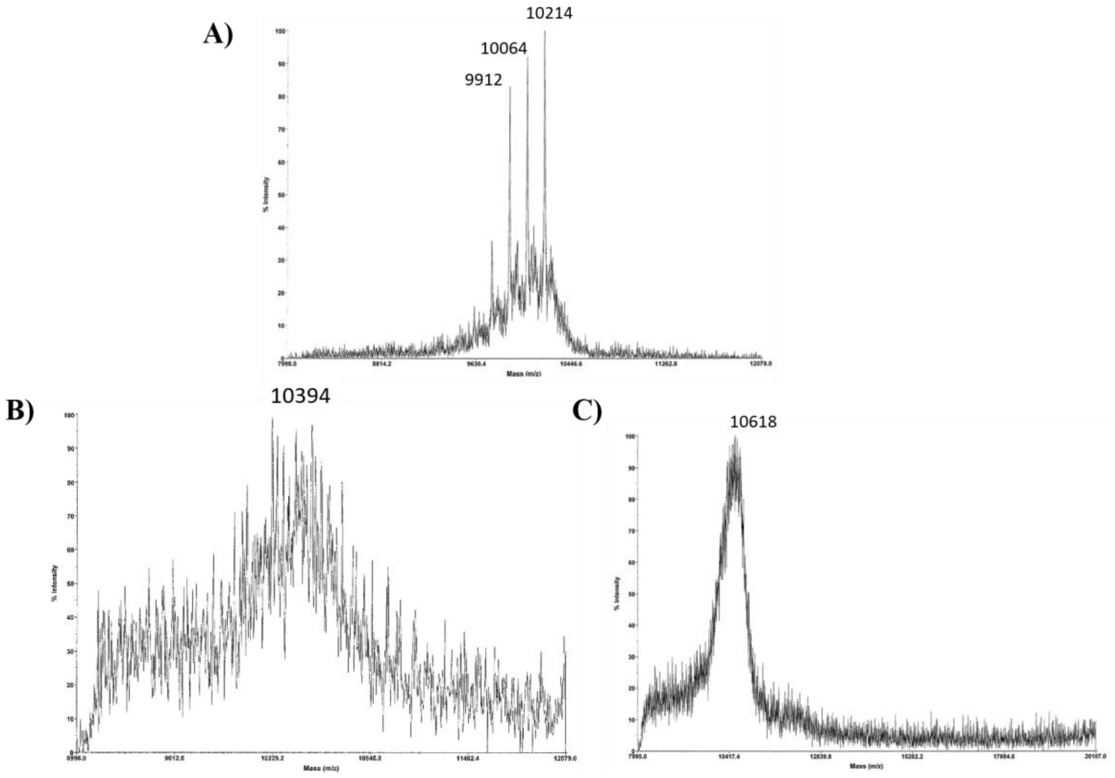

Figure S9: MALDI-TOFF characterized of the S901-Cy5 oligonucleotide and its Hytacs conjugates. A) S901-Cy5, B) Adamantyl-S901-Cy5 and C) Boc$_3$Arg-S901-Cy5.

7. CELLULAR ASSAYS WITH APTAMER-HYTACS

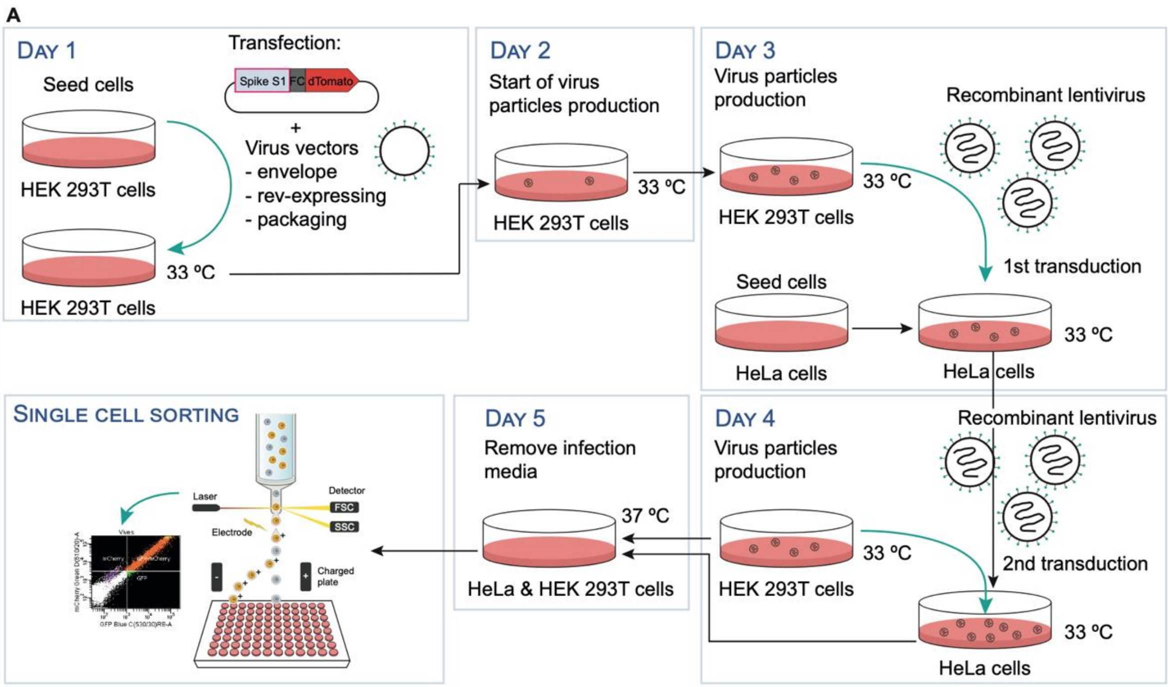

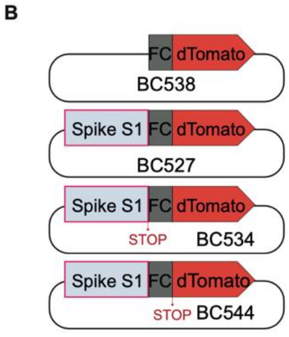

C

Cell line	Vector	FACS	growing / seeded	% survival	+ clones
HEK293T	BC538	60%	23 / 48	48 %	14
	BC527	38.2%	17 / 48	35 %	10
	BC534	34.2%	19 / 48	40 %	5
	BC544	38.5%	12 / 48	25 %	4
HeLa	BC538	7%	10 / 24	42 %	2
	BC527	0.9%	10 / 24	42 %	4
	BC534	55.6%	10 / 24	42 %	0
	BC544	53.5%	13 / 24	51 %	0

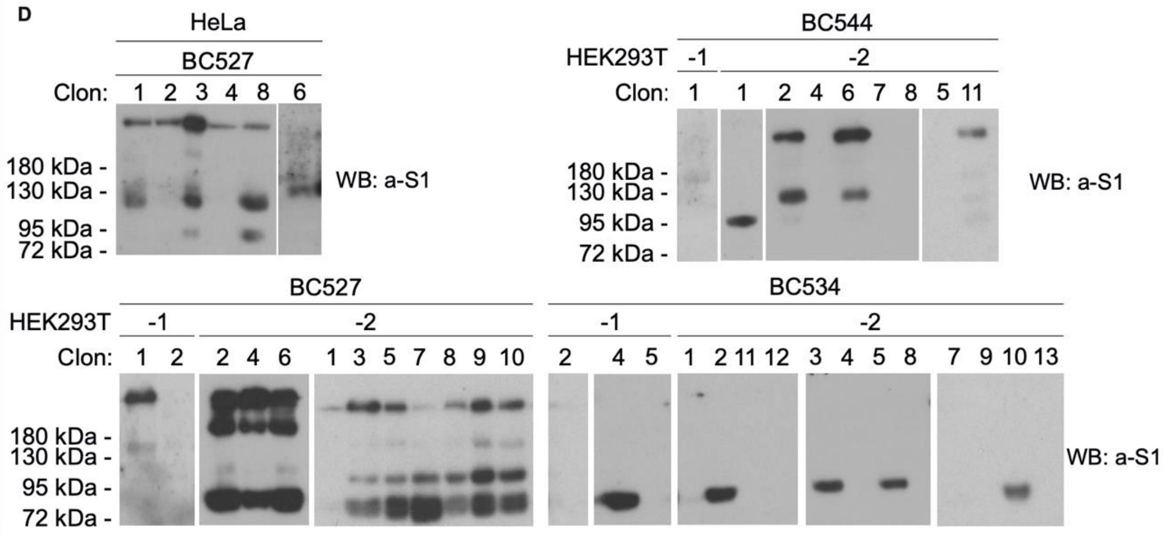

Figure S10. Generation of Cell lines expressing stable S1. A. Schematic representation of the process for generation of human cells stably expressing S1 protein, used in this study. **B**. Schematic representation of the vectors used in this study. **C**. Table showing efficiency indicators and number of clones obtained in each cell line for each vector. **D**. Western blots showing the expression of S1 in the clones selected.

--

Figure S11. Cell viability of cells treated with aptamers:

A. Assessment of HeLa and HeLa527 cell viability in the presence of aptamers A01 and A04

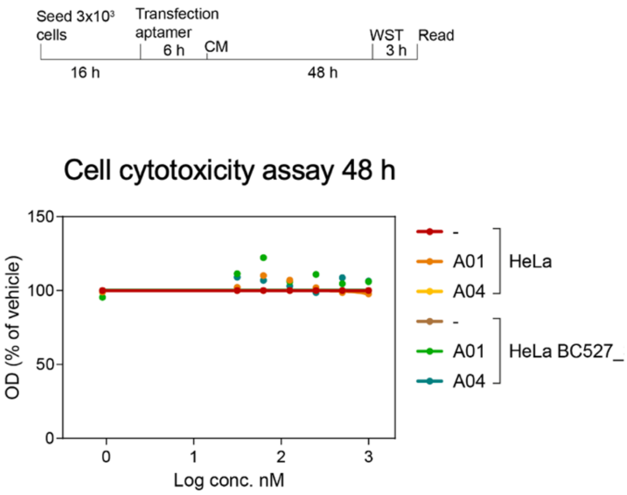

B. Table showing percentages of alive and dead cells upon treatment with compounds.

Cell line	Compound	Alive (%)	Dead (%)
HeLa538	-	99.8	0.19
HeLa538	A08	97.0	2.47
HeLa538	A09	97.4	2.12
HeLa538	A10	98.3	1.18
HeLa527	-	99.6	0.41
HeLa527	A08	93.8	5,12
HeLa527	A09	97.7	1.80
HeLa527	A10	98.5	1.16
HEK293T-538	-	98.7	1.07
HEK293T-538	A08	93.8	5.41
HEK293T-538	A09	94.3	5.11
HEK293T-538	A10	97.4	2.14
HEK293T-527	-	97.6	2.21
HEK293T-527	A08	95.7	3.73
HEK293T-527	A09	94.6	4.78
HEK293T-527	A10	98.3	1.51

C. Graphics showing the populations of cells analyzed by cytometry (as shown in B).

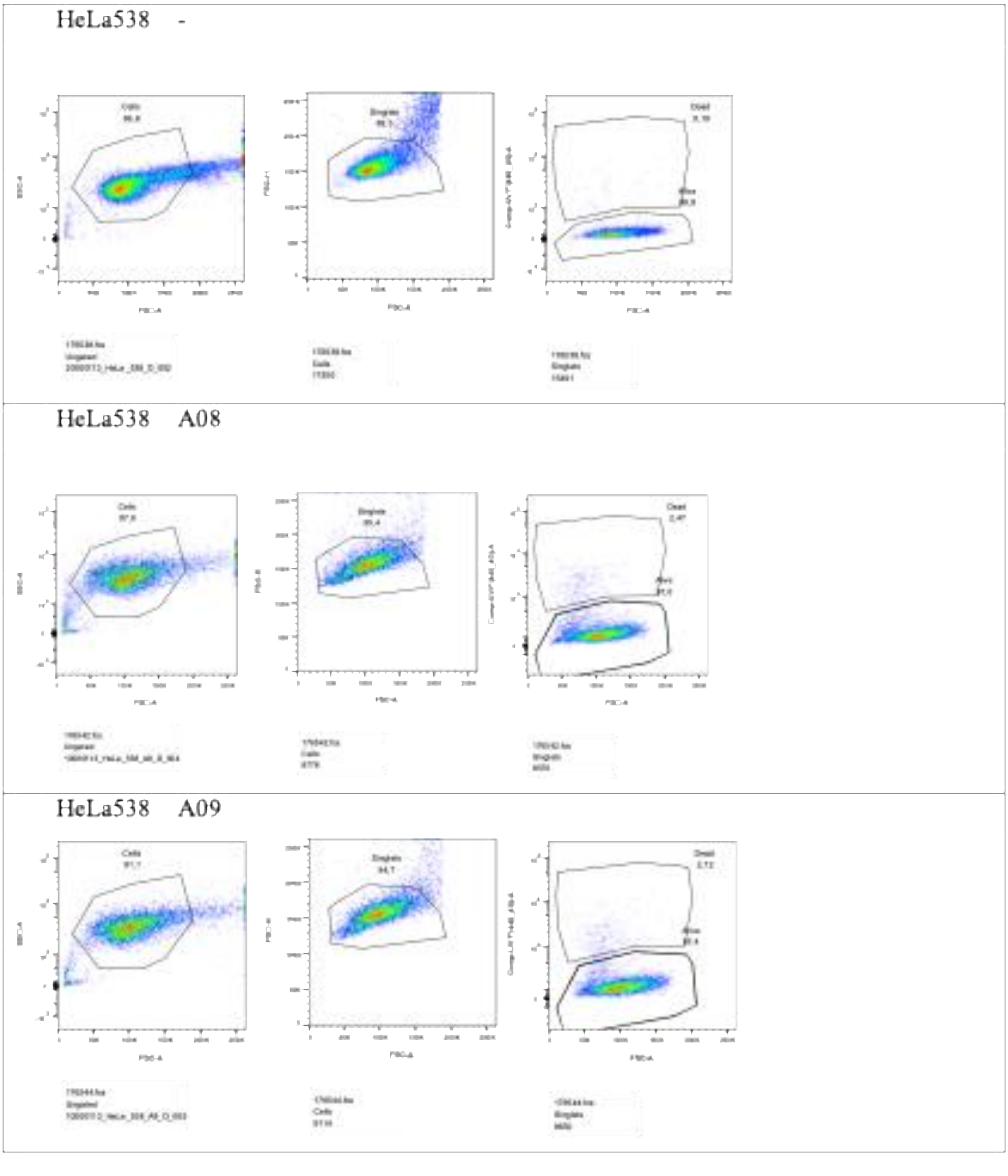

HeLa538 A10

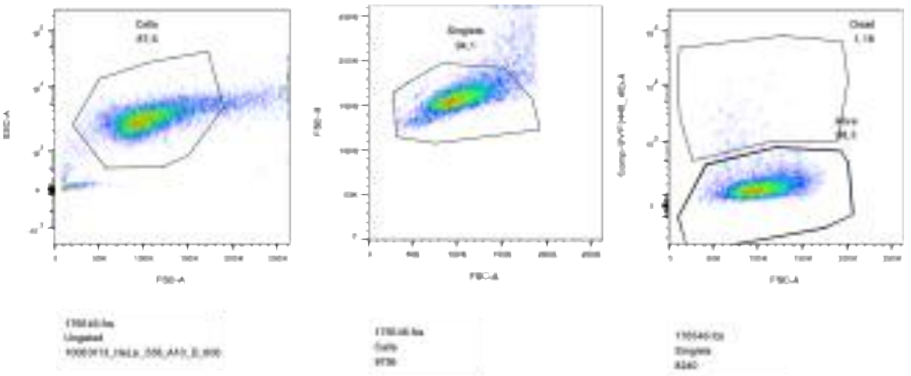

HeLa527 -

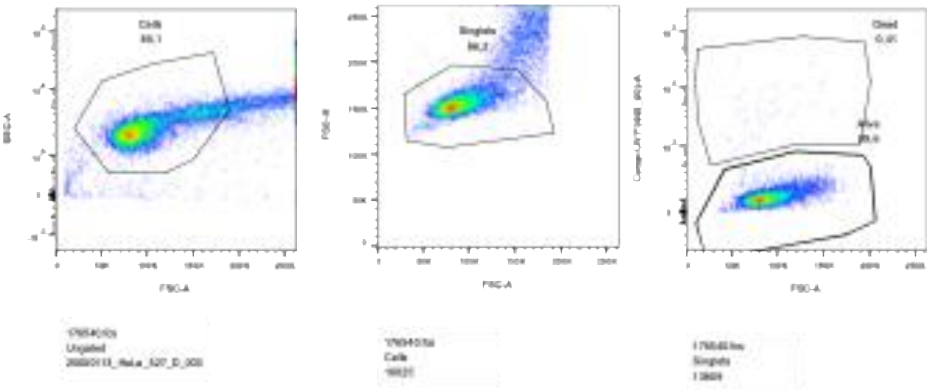

HeLa527 A08

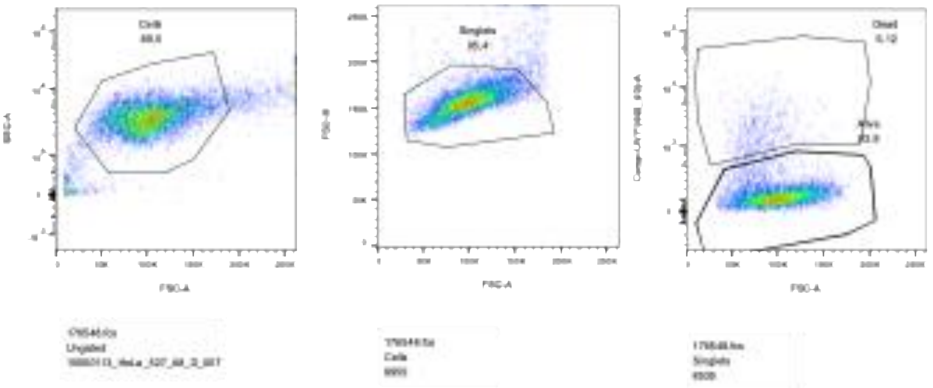

HeLa527 A09

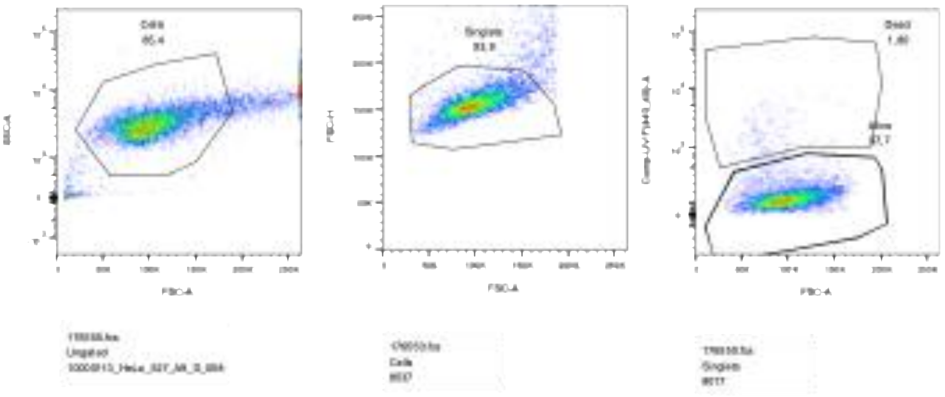

HeLa527 A10

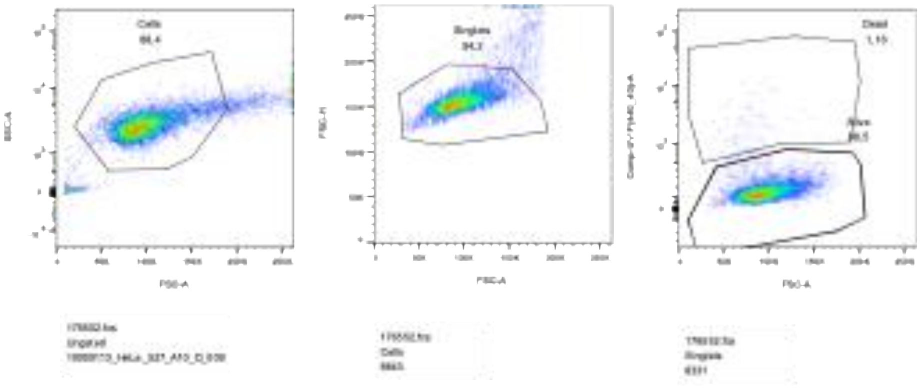

HEK293T-538 -

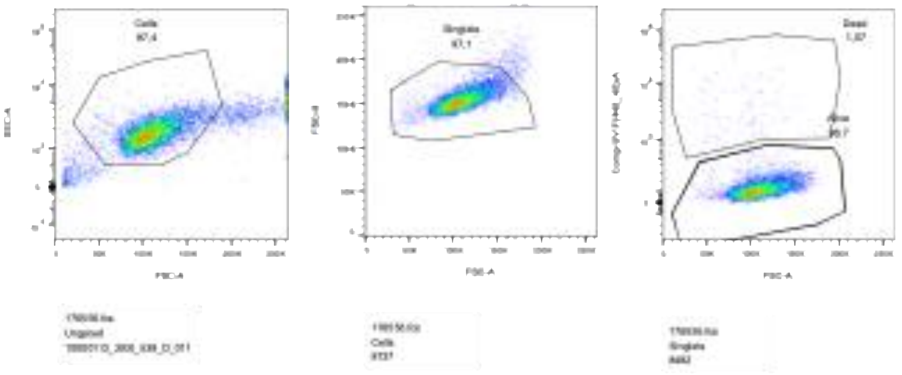

HEK293T-538 A08

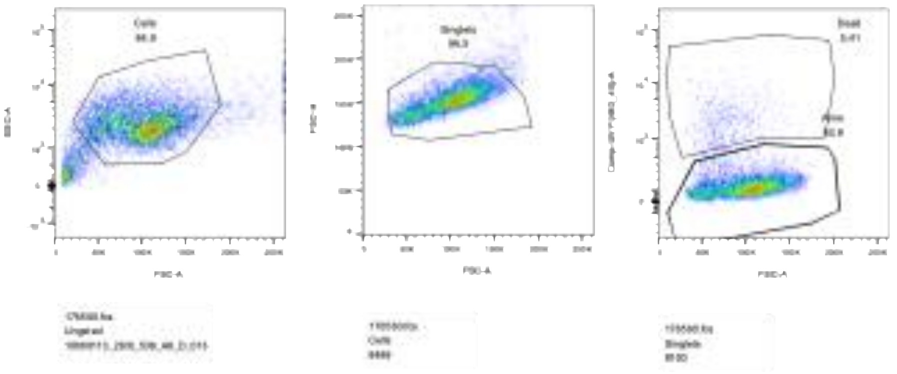

HEK293T-538 A09

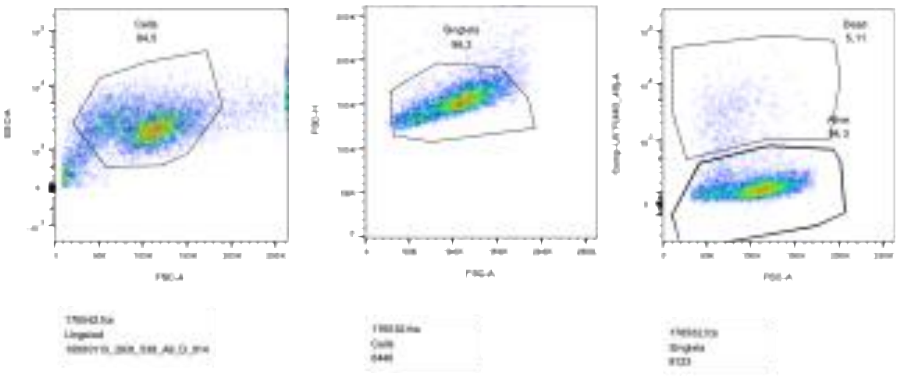

HEK293T-538 A10

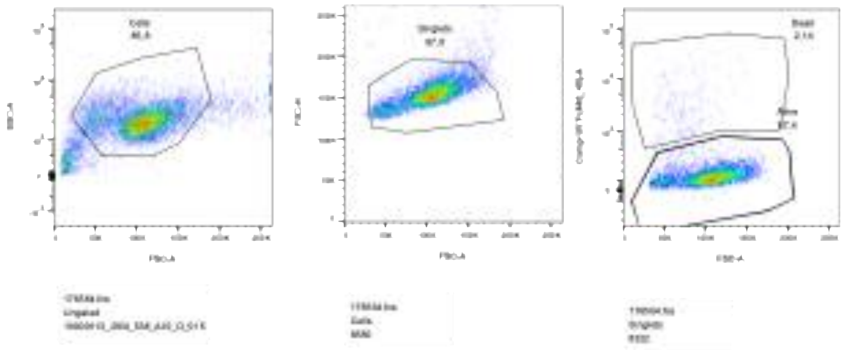

HEK293T-527 -

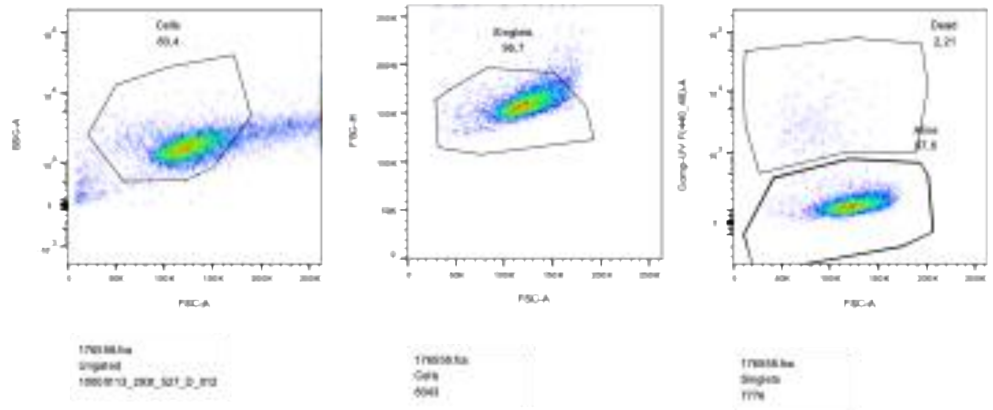

HEK293T-527 A08

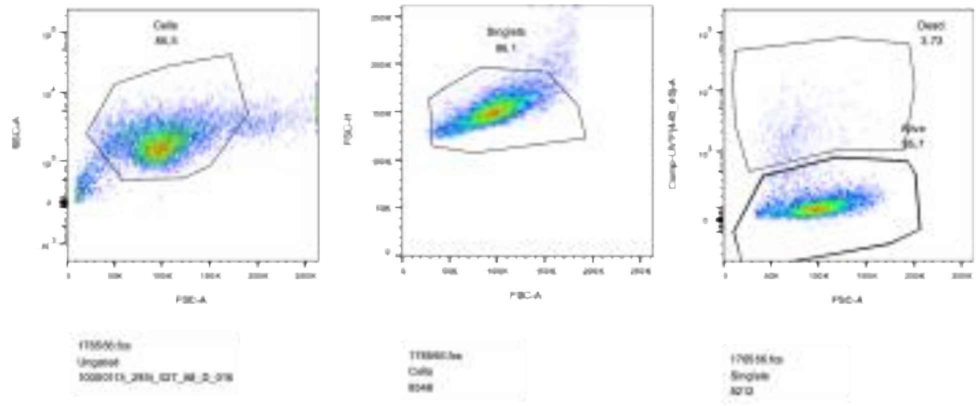

HEK293T-527 A09

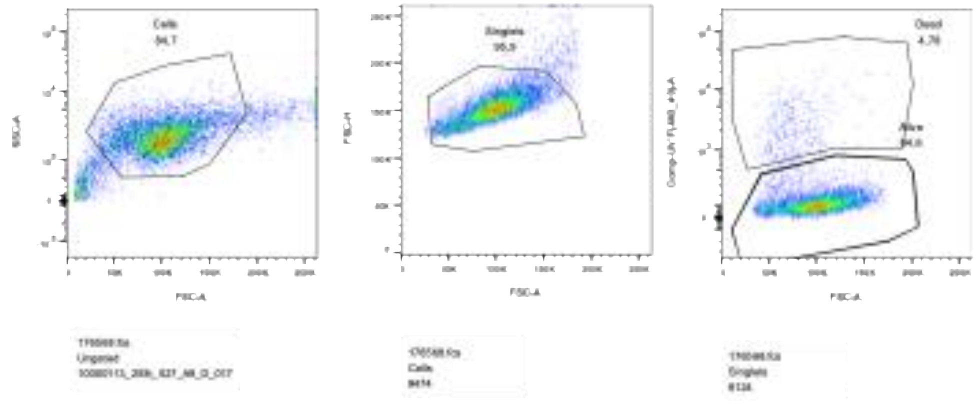

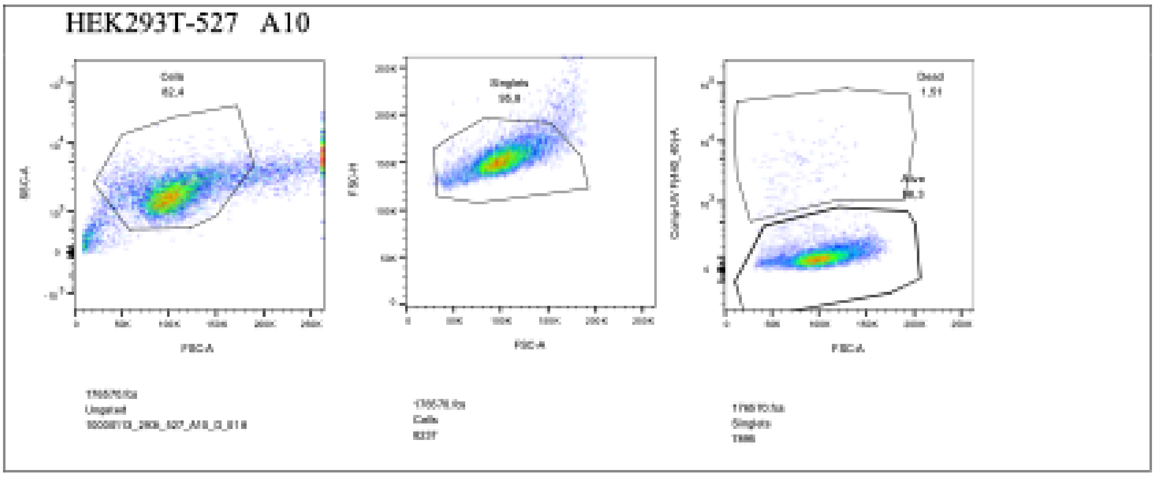

Figure S11. Cell viability of cells transfected with Cy5-labeled aptamers (A08, A09 and A10).

A. Assessment of HeLa and HeLa527 cell viability in the presence of aptamers A01 and A04. For that purpose, cells were plated at a density of 3,000 cells/well in a 96-well plate. Upon 16 h of culture, cells were treated with the indicated doses of compounds (concentration range, 30 nM -1000 nM). Media were changed 6 hours after transfection, and treatment was performed for a total of 48 hours. WST-8 reagent (Abcam) was added to cell culture media 3 h prior to reading absorbance at 440 nm with a SYNERGY HTX Absorbance microplate reader (BioTek).

B. Cell viability of cells transfected with Cy5-labeled aptamers (A08, A09 and A10). The Table shows the percentages of alive and dead cells after transfection.

C. Graphics showing the populations of cells analyzed by cytometry (as shown in B).

Representation of the 16 conditions of sorting of alive and dead cells carried out by flow cytometry, shown in panel **B**. Values of alive and dead cell do not sum 100% because they are not exactly complementary windows.

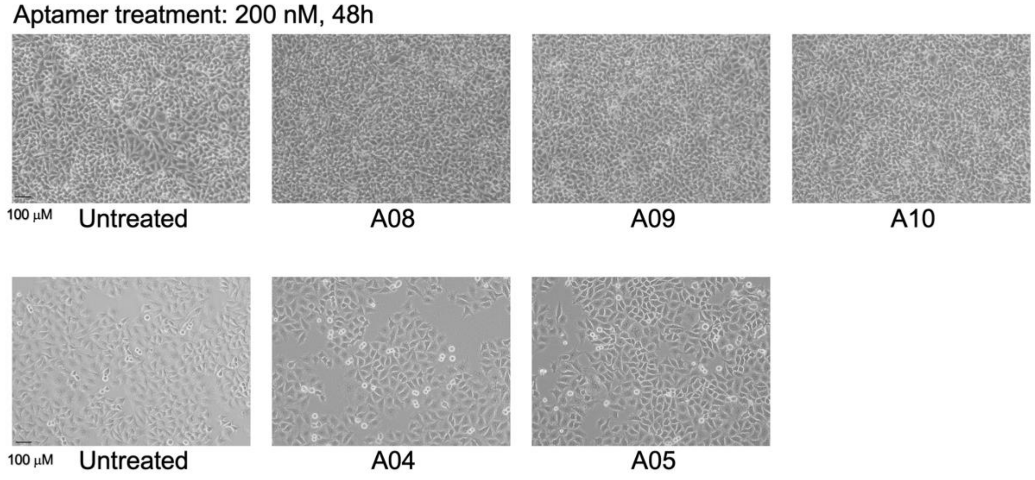

Figure S12. Microscopy images of cells treated with aptamer-Hytacs. Brightfield images of treated HeLa cells, including untreated control, non-conjugated aptamers control (A08 and A04) and treatments with aptamer-Hytacs (A09, A10 and A05).

Table S1. MALDI-TOFF characterization

Sequence	$Mass_{Cal}$	$Mass_{exp}$
NH_2-T_8	2544.1	2545.1
Ada-NH-T_8	2697.9	2707.1
L-Ala(Ada)-NH-T_8	2749.7	2740.0
Boc_2Arg-NH-T_8	2925.2	2905.1
[a]Boc_3-Arg-NH-T_8	2975	3006.9 (M+Na+), 2906.8 (M+Na+-1 Boc group)[c], 2806.7 (M+Na+-2 Boc groups)[c]
[b]Boc_3-Arg-NH-T_8	2975	3006 (M+ Na), 2906.3 (M+ Na+-1 Boc group)[c], 2806.5 (M+ Na+-2 Boc groups)[c]
S901-Cy5	10230	10214.6
Adamantyl-S901-Cy5	10394	10493.8, 10229
Boc_3-Arg-S901-Cy5	10675	10618 (M+ 2Na -1 Boc group)[a]
S901-HEX-Biotin	10596	10442
Adamantyl-S901-HEX-Biotin	10759	10755.7, 10584, 10464
Boc_2Arg-S901-HEX-Biotin	10951	nd
S701-HEX-Biotin	17853	nd
Adamantyl-S701-HEX-Biotin	18016	nd
Boc_2Arg-S701-HEX-Biotin	18224	nd

[a]Coupling agent PyBOP and [b]coupling agent HATU. [c]a Boc group weights 99 mass units, HEX: hexaethyleneglycol, Biotin is the biotin-tetraethylenglycol derivative (biotine-TEG), nd not detected, S901(30mer). S702(53mer).
